# Cu-Doped Cs_3_Sb_2_Cl_9_ Nanocrystals: Revisiting the Low Bandgap of Cs_2_CuSbCl_6_ Double Perovskites

**DOI:** 10.1021/acsmaterialslett.5c01043

**Published:** 2025-10-10

**Authors:** Simone Virga, David F. Macias-Pinilla, Nicola Dengo, Federica Bertolotti, Alessandro Longo, Fei He, Quinten A. Akkerman, Francesco Giannici

**Affiliations:** † Department of Physics and Chemistry, 18998 University of Palermo, viale delle Scienze, 90128 Palermo, Italy; ‡ Department of Science and High Technology and Total Scattering Laboratory (To.Sca.Lab), 19045 University of Insubria, via Valleggio 11, 22100 Como, Italy; § BM16-FAME Beamline, The European Synchrotron Research Facility, 71 Avenue des Martyrs, 38000, Grenoble, France; # ISMN-CNR, UOS Palermo, via Ugo la Malfa 156, 90143, Palermo, Italy; ∥ Chair for Photonics and Optoelectronics, Nano-Institute Munich, Department of Physics, 9183 Ludwig-Maximilians-Universität (LMU), Königinstraße 10, 80539 Munich, Germany

## Abstract

Lead-free halide double perovskite Cs_2_CuSbCl_6_ nanocrystals have recently been reported to have a low bandgap of 1.66 eV. In this work, we show that the optical absorption spectra and X-ray diffraction patterns previously attributed to Cs_2_CuSbCl_6_ can rather be explained with Cs_3_Sb_2_Cl_9_:Cu: X-ray absorption spectroscopy identifies [CuCl_3_]^−^ trigonal pyramids, with Cu^2+^ possibly replacing two Cs^+^ sites. The broad low-energy optical absorption is then assigned to localized electronic transitions at copper dopants within the Cs_3_Sb_2_Cl_9_ lattice, which do not affect the wide bandgap. Ab initio calculations suggest that Cs_2_CuSbCl_6_ is thermodynamically unstable with respect to decomposition to Cs_3_Sb_2_Cl_9_, in line with the low reproducibility of Cs_2_CuSbCl_6_ observed in its synthesis.

## Graphical abstract

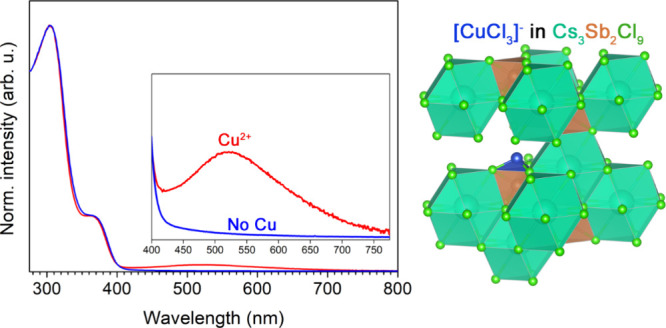

Lead halide perovskites, either inorganic or hybrid, are among the most studied compounds in recent materials chemistry research, due to their outstanding optoelectronic properties and their potential applications in photovoltaics, thermoelectrics, as classical light sources such as light-emitting diodes and liquid crystal displays, as quantum light sources, lasing materials, and radiation detection.^1,2^ Pb^2+^ sits at an optimum combination of factors as what concerns size, electronic configuration, and oxidation state, but high toxicity, and low chemical stability of hybrid perovskites give at the same time the incentive to find alternative compositions.^3^ Substitution with isovalent ions (Ge^2+^ or Sn^2+^) preserves both stoichiometry and the 3D structure, but with low redox stability.^4^

The heterovalent replacement of Pb^2+^, on the other hand, leads to several different perovskite-related structures,^5^ among which double perovskites (*elpasolites*) are the most studied. These cubic halide double perovskites (HDP) involve the alternating arrangement of a monovalent and a trivalent cation in the Pb^2+^ sites, resulting in A_2_BB’X_6_ compositions.^6,7^ Most HDP have good chemical stability, and their optoelectronic properties were shown to be highly tunable through doping and alloying.^8–11^ Even if still uncompetitive with lead-based counterparts for photovoltaics, HDP have proven interesting, especially in the form of nanocrystals (NCs)^12^ for e.g. light emission,^13–15^ light detection,^16^ X-ray detection,^17^ solar-to-heat conversion^18^ or photocatalysis.^19^ Compared to their bulk counterparts, HDP NCs may exhibit markedly different optical and structural properties,^20,21^ and greater bandgap tunability, but generally require careful surface chemistry control to prevent degradation and preserve the optoelectronic properties.^6,22^

A general theoretical appraisal on Cu HDP showed that their lower bandgap compared to Ag HDP comes at a cost, since AgCl_6_ octahedra are stable while Cu^+^ prefers a lower coordination arrangement, due to the Cu 3d states in the valence band maximum (VBM) being at a higher energy than Ag 4d.^23^ Indeed, Wang et al. recently proposed that Cs_2_CuSbCl_6_ NCs are metastable.^24^ In fact, a recent review on copper-containing perovskites and perovskite-like structures (2D-0D) highlighted that Cu HDP remain mainly at the theoretical level due to stability concerns.^25^ In this generally unfavorable landscape on Cu HDP, the report of Cs_2_CuSbCl_6_ NCs with a low bandgap of 1.66 eV in 2020 understandably sparked a widespread interest.^26^ This was in fact the smallest bandgap reported so far in an HDP, and Cs_2_CuSbCl_6_ has been the object of renewed research efforts in the last five years: it was used as an archetype HDP to illustrate topological states^27^ and band structure,^28,29^ featured prominently in HDP reviews,^25,30–32^ and even in photovoltaic device simulations.^33,34^

In this work, we show that both the optical absorption spectra and the X-ray diffraction (XRD) pattern attributed to Cs_2_CuSbCl_6_ are rather due to Cu-containing Cs_3_Sb_2_Cl_9_, where localized ligand-to-metal charge transfer (LMCT) and d-d electronic transitions in [CuCl_3_]^−^ clusters are responsible for the wide optical absorption centered at 530 nm. Ab initio calculations are eventually used to support the observation that Cs_2_CuSbCl_6_ is thermodynamically unstable toward decomposition, and that the presence of Cu^2+^ centers in Cs_3_Sb_2_Cl_9_ does not lead to an overall lower bandgap.

To validate the Cs_2_CuSbCl_6_ NCs preparation, we followed the synthesis method reported in ref^26^ obtaining a similar purple colloidal suspension. The resulting XRD pattern indicates the formation of the trigonal Cs_3_Sb_2_Cl_9_ phase instead of cubic Cs_2_CuSbCl_6_ (Figure 1a and S1), which features very similar Bragg peaks. Repeated batches of the same sample sometimes also resulted in the formation of a minor fraction of Cs_2_CuSbCl_6_ (Figure S2), which did not change the optical absorption in the visible range (Figure S3). When Cs and Sb react in the absence of Cu, two polymorphs of Cs_3_Sb_2_Cl_9_ are formed (about 30% trigonal and 70% orthorhombic) (Figure S4). When Cu is present, the sample is single-phase with just the trigonal polymorph. Since Cu directs the crystallization process toward this latter polymorph, it needs to be in the crystal lattice of Cs_3_Sb_2_Cl_9_ (see Figure S5–S12 for the Rietveld refinements). The expected XRD patterns of trigonal Cs_3_Sb_2_Cl_9_ and of the Cs_2_CuSbCl_6_ structure proposed previously (see SI of ref^26^) are similar, which explains the misattribution of the Bragg peaks in ref.^26^ However, the Cs_2_CuSbCl_6_ structure proposed previously, with a cubic lattice parameter of 10.757 Å, involves very long Cu–Cl bonds at 2.61 Å (see Figure S13 for average Cu–Cl bond lengths in the literature). Most importantly, the proposed unit cell volume would be larger than the isostructural Cs_2_AgSbCl_6_ (10.664 Å),^35^ despite Ag^+^ being in fact much larger than Cu^+^ (1.15 vs 0.77 Å). Our density functional theory (DFT) calculations confirm a monotonic reduction in the lattice parameter upon gradual substitution of Ag^+^ by Cu^+^, eventually reaching a smaller value for Cs_2_CuSbCl_6_, in agreement with ionic size considerations (Figure S14). While no XRD data modeling was provided, Karmakar et al. showed that Cu^2+^ contracts the lattice parameter of polycrystalline Cs_2_AgSbCl_6_ up to 10% substitution, consistently with a lower ionic radius of Cu^2+^ compared to Ag^+^.^36^ Wang et al. reported metastable Cs_2_CuSbCl_6_ nanocrystals using modified ligand-assisted reprecipitation. Although the reported XRD data matched with a cubic perovskite symmetry, no lattice parameters were provided. In fact, they found a theoretically predicted structure with [CuCl_3_]^2–^ and [SbCl_6_]^3–^ polyhedra to be the most stable for Cu^+^ and Sb^3+^.^24^

**Figure 1:**
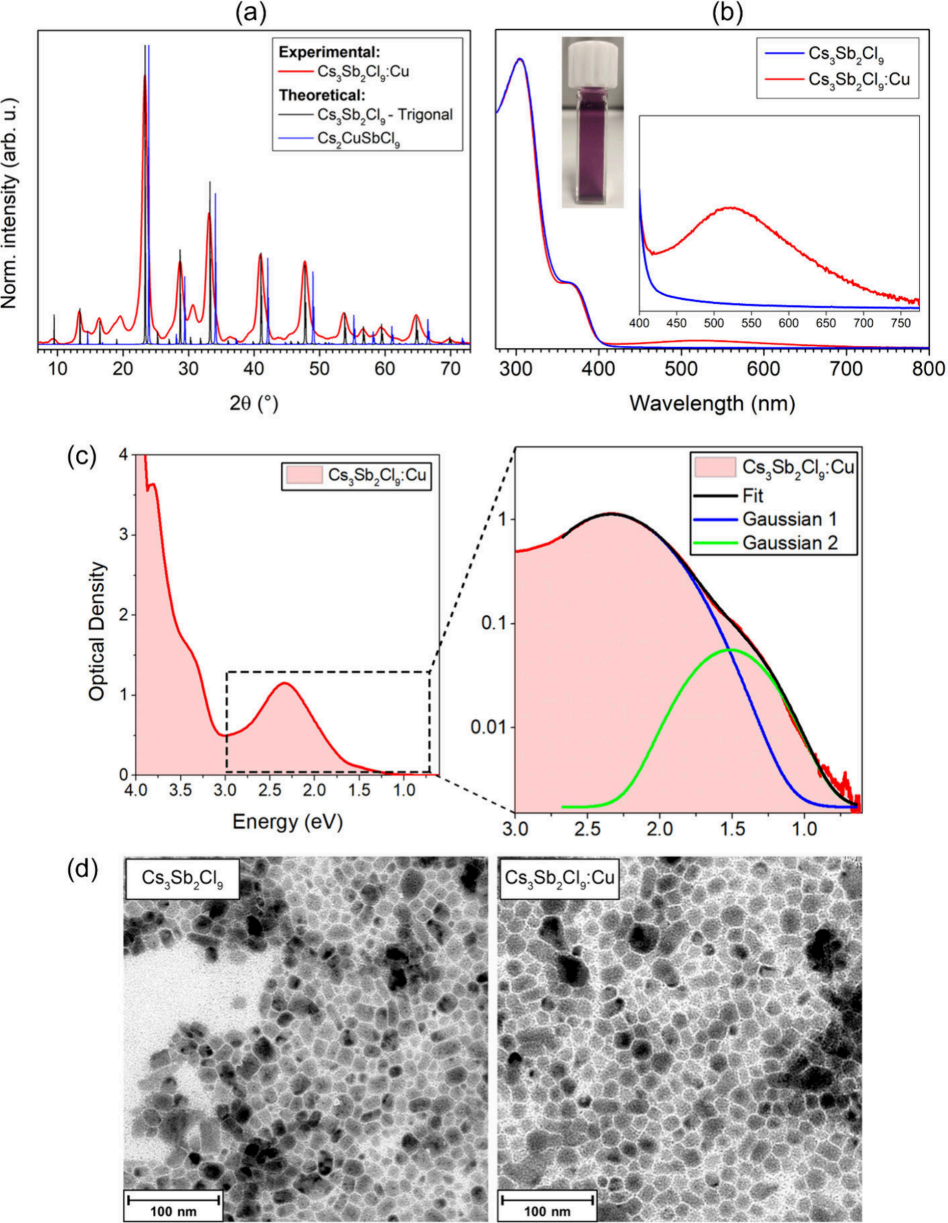
(a) XRD pattern (for λ = 1.5418 Å) of the sample Cs_3_Sb_2_Cl_9_:Cu compared with simulations of the XRD patterns of trigonal Cs_3_Sb_2_Cl_9_ and Cs_2_CuSbCl_6_. (b) UV–vis absorption spectrum of Cs_3_Sb_2_Cl_9_:Cu compared with Cs_3_Sb_2_Cl_9_. (c) UV–vis–NIR absorption spectrum of Cs_3_Sb_2_Cl_9_:Cu. (d) TEM images of Cs_3_Sb_2_Cl_9_ and Cs_3_Sb_2_Cl_9_:Cu.

The optical absorption spectrum of Cs_3_Sb_2_Cl_9_:Cu (Figure 1b) displays a broad absorption peak centered around 530 nm, with a full width at half-maximum of 170 nm, in agreement with the literature.^26^ Such a broad absorption peak, attributed to the bandgap of Cs_2_CuSbCl_6_,^26^ does not resemble a fundamental bandgap, which should not result in an absorption peak, nor an exciton in a semiconductor.^37^ On the other hand, such broad absorption features are rather common for LMCT and d–d transitions in coordination complexes. For instance, Cu–Cl complexes exhibit various charge-transfer transitions from 350 to 635 nm.^38,39^ In addition to the broad absorption at 530 nm, the Cs_3_Sb_2_Cl_9_:Cu NCs also exhibit two absorption peaks at 304 and 361 nm:^12,40,41^ these were not shown in ref 26 due to the spectrum being cut below 400 nm. To investigate the nature of these lower-wavelength absorption peaks, further reactions were conducted with different ratios of Cs:Cu:Sb, namely 1:0:1, 1:1:0 and 0:0:1 (Figure S15). The peaks below 400 nm are observed with a Cs:Cu:Sb ratio of 1:0:1, consistent with the direct and indirect band gaps of α- and β-Cs_3_Sb_2_Cl_9_ at 2.85–2.9 eV.^42^ The broad absorption band around 2.3 eV/530 nm is due to copper instead, and can be tentatively attributed to the incorporation of Cu into trigonal Cs_3_Sb_2_Cl_9_.

Extending the analysis into the near-infrared (NIR) region, although the signal is relatively weak, an absorption shoulder can be discerned (Figure 1c), at 1.5 eV/820 nm, with an intensity around 5% of that of the main peak at 2.3 eV/530 nm. The shape and intensity ratio of these bands are consistent with LMCT and d-d transitions,^38,43,44^ and they can only be due to Cu^2+^ ions in a d^9^ configuration, as they are forbidden in d^10^ Cu^+^ ions, providing additional evidence against the attribution of such band to the Cs_2_CuSbCl_6_ double perovskite. The only allowed localized electronic transition in Cu^+^ (3d → 4s/p) is expected at 300 nm or lower.^45^

TEM images (Figure 1d) confirm the effect of Cu in directing the growth Cs_3_Sb_2_Cl_9_, resulting in a sharper size distribution and smaller average size (20 nm vs 23 nm). (Figure S16). Increasing the Cu loading always results in the formation of trigonal Cs_3_Sb_2_Cl_9_:Cu with different intensity of the 530 nm band relative to the 304 and 361 nm absorption transitions (Figure S17), further proving that this band is due to Cu^2+^ centers in the structure. Once the Cu:Sb ratio reaches 2:1, the absorption spectrum changes radically, since a new phase is formed, which XRD identifies as Cs_2_CuCl_4_ (Figure S18).

The Cu K-edge X-ray Absorption Near Edge Structure (XANES) spectra of Cs_3_Sb_2_Cl_9_:Cu and Cs_2_CuCl_4_ are shown in Figure 2a. Both samples exhibit a pre-edge peak at 8977 eV, attributed to a 1s → 3d electronic transition, which is dipole-forbidden and only occurs in Cu^2+^, with the lowest intensity for D_4h_ coordination geometry and the highest for D_2d_.^46^ In contrast, Cu^+^ species only exhibit an allowed 1s → 4p transition around 8983–8984 eV (8986–89 eV for Cu^2+^), which is not evident in the present spectra.^47,48^ This corroborates that copper is in the +2 oxidation state in Cs_3_Sb_2_Cl_9_:Cu. Such a finding is again in contrast with a Cs_2_CuSbCl_6_ double perovskite, requiring Cu^+^.

**Figure 2:**
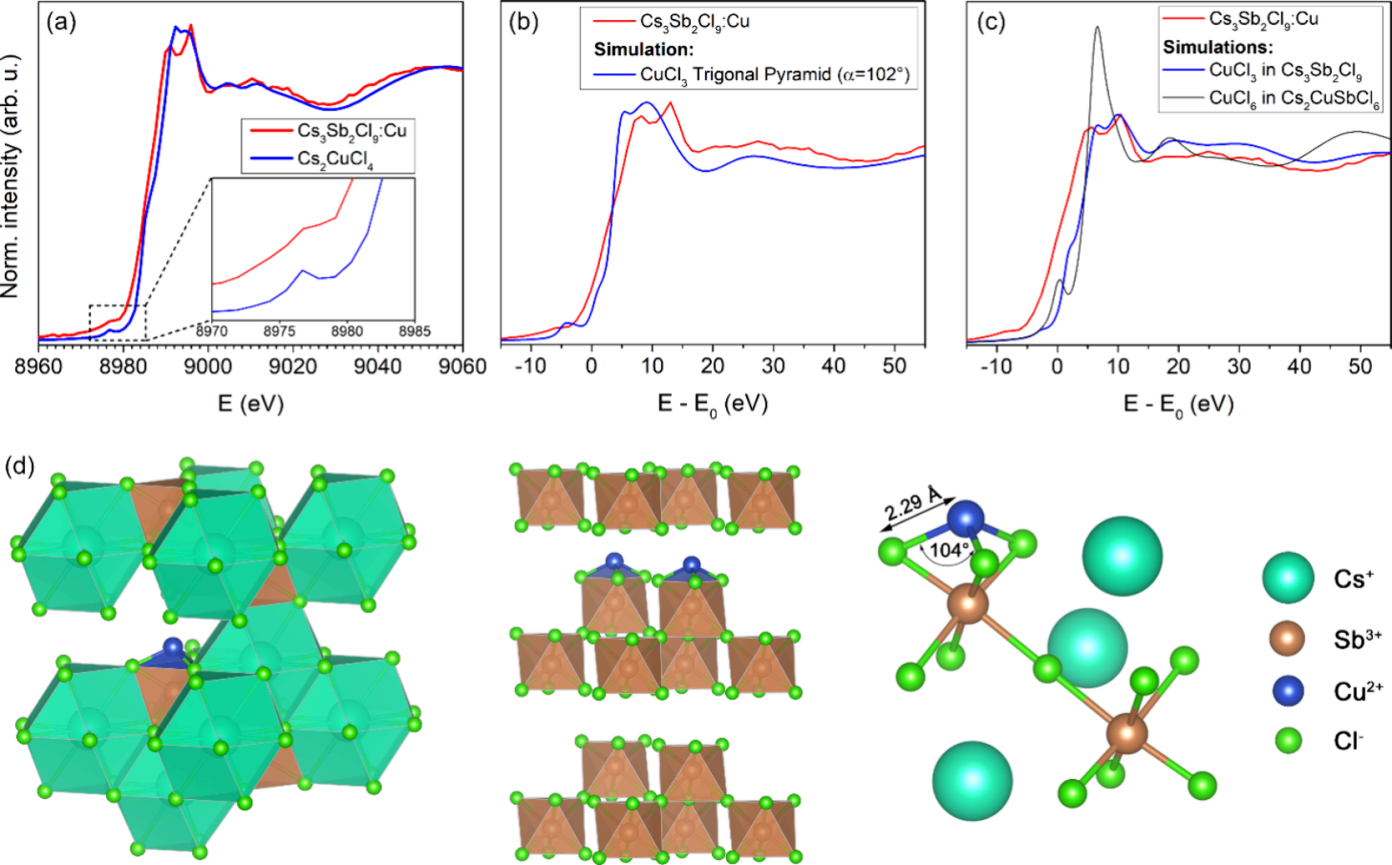
(a) XANES spectra of the Cu K-edge of Cs_3_Sb_2_Cl_9_:Cu and experimental Cs_2_CuCl_4_. (b–c) XANES spectra for the Cu K-edge of Cs_3_Sb_2_Cl_9_:Cu compared with (b) XANES simulation of CuCl_3_ with α=102° and (c) XANES simulations of trigonal pyramidal CuCl_3_ in Cs_3_Sb_2_Cl_9_ and octahedral CuCl_6_ in Cs_2_CuSbCl_6_. (d) Possible location of a trigonal pyramidal [CuCl_3_]^−^ center in the Cs_3_Sb_2_Cl_9_ lattice (see Figure S24 for possible locations).

Cai et al. reported the synthesis of Cs_4_CuSb_2_Cl_12_ layered perovskite NCs featuring Cu­(II)­Cl_6_ octahedra, confirmed by XRD and TEM, and attributed a 530 nm broad absorption to a direct band gap.^16^ While the XRD patterns of Cs_4_CuSb_2_Cl_12_ and our Cs_3_Sb_2_Cl_9_:Cu sample are clearly different, the UV–vis absorption spectra are remarkably similar. In particular, also in the case of Cs_4_Cu_*x*_Ag_2–2*x*_Sb_2_Cl_12_ the intensity of the 530 nm band increases with Cu loading, suggesting it is also due to localized transitions at the Cu^2+^ site.

From the extended X-ray absorption fine structure (EXAFS) analysis (Figure S19), Cu is coordinated by three Cl atoms at 2.29 Å in Cs_3_Sb_2_Cl_9_:Cu, significantly shorter than the Cu–Cl distance of 2.61 Å in the HDP structure proposed in ref 26. The XANES simulation of tetrahedral CuCl_4_ clusters reproduces the experimental data of the experimental Cs_2_CuCl_4_, validating the model (Figure S20). Since the experimental spectra for Cs_2_CuCl_4_ and Cs_3_Sb_2_Cl_9_:Cu are very similar in the EXAFS region (as the coordinating atoms are the same), but differ significantly at the absorption edge, this suggests the Cu coordination geometry is not tetrahedral in Cs_3_Sb_2_Cl_9_:Cu.

To gain further insight into the local structure of copper, XANES simulations of various trial CuCl_n_ clusters were compared with Cs_3_Sb_2_Cl_9_:Cu, and the best agreement was found with a trigonal CuCl_3_ complex (Figure S21). Further simulations were performed varying the Cl–Cu–Cl dihedral angle from 120° (flat geometry) to 90° (trigonal pyramidal geometry). Decreasing the angle between 102 and 110° gives the best agreement with the experiment (Figure S22), suggesting a distorted, trigonal pyramidal coordination environment. Finally, simulations of this CuCl_3_ trigonal pyramid were performed by adjusting the electronic screening values on the Cu photoabsorber (Figure S23), and the best simulation is eventually reported in Figure 2b. This result provides strong evidence that in Cs_3_Sb_2_Cl_9_:Cu copper is coordinated by three chloride ligands in a distorted trigonal pyramidal geometry, with a Cl–Cu–Cl angle around 104° and Cu–Cl bond lengths of about 2.29 Å. Such a coordination environment can be achieved in the trigonal Cs_3_Sb_2_Cl_9_ lattice if Cu resides e.g. an empty Cs site with significant off-centering.

Cu^2+^ is much smaller than Cs^+^, but a similar coordination was described by Kaiukov et al.^49^ in the perovskite-related structure Cs_3_Cu_4_In_2_Cl_13_, with a polynuclear cluster of four tetrahedrally coordinated Cu in place of Cs. Similar arrangements, with less than four CuCl_3_ units, can also accommodate in the Cs cavity with appropriate distortion. Further confirmation that Cu^2+^ does not sit in an octahedral environment is provided by the XANES of CuCl_6_ octahedra (Figure 2c), which result in a completely different simulated spectrum, also ruling out the possible Sb/Cu substitution in Cs_3_Sb_2_Cl_9_. One possible way to allocate the [CuCl_3_]^−^ unit in the Cs_3_Sb_2_Cl_9_ lattice can be obtained by substituting two Cs^+^ ions with one Cu^2+^ ion, resulting in [CuCl_3_]^−^ in-between the Sb octahedral layers, in which the Cu–Cl distances and angles are all in agreement with the EXAFS and XANES evidence (Figure 2c and 2d).

Complementary evidence was collected also from the Sb K- and L_3_-edges, and the Cl K-edge. The Sb K- (Figure S25) and L_3_-edge (Figure 3a) spectra have little difference between the two samples with and without Cu: the subtle variation in the postedge region is attributable to the absence of the orthorhombic polymorph induced by the incorporation of Cu. The XANES simulations of trigonal Cs_3_Sb_2_Cl_9_ show a good agreement with the experimental Cs_3_Sb_2_Cl_9_, both at the Sb K- and L_3_-edges (Figure S26), confirming at the local level what is observed by XRD on the long-range. Similarly, the experimental Sb L_3_-edge spectrum of Cs_3_Sb_2_Cl_9_:Cu (Figure 3b) does not match the simulations of the double perovskite Cs_2_CuSbCl_6_, but is instead well reproduced by the simulations of the trigonal Cs_3_Sb_2_Cl_9_ (similarly for Sb K-edge, see Figure S26). This provides further confirmation on the coordination state of all Sb atoms.

**Figure 3:**
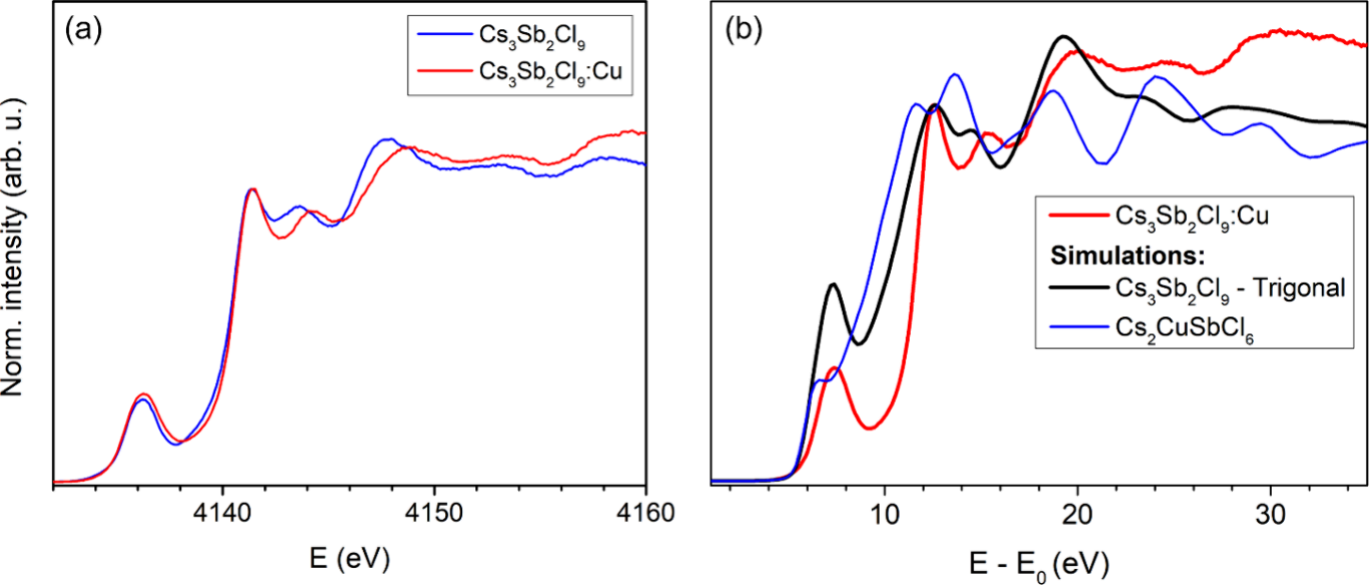
(a) XANES spectra of experimental Cs_3_Sb_2_Cl_9_ and Cs_3_Sb_2_Cl_9_:Cu for Sb L_3_-edge. (b) Sb L_3_-edge of Cs_3_Sb_2_Cl_9_:Cu, compared with trigonal Cs_3_Sb_2_Cl_9_ and Cs_2_CuSbCl_6_.

The Cl K-edge probes all the bonds between chlorine and metals: the pre-edge transition observed at 2819 eV represents the transition from Cl 1s to hybridized empty states of Cl 3p and Cu 3d, and it then is assigned to Cu^2+^-Cl by comparison with literature data on CuCl_2_ and CuCl.^50^ The intensity of this peak reflects the covalent character of the metal-chloride interaction, and it is notably absent in Cs_3_Sb_2_Cl_9_ (Figure S27) where the Sb–Cl interaction is ionic.^46^

To rationalize the experimental absence of the Cs_2_CuSbCl_6_ perovskite phase, we performed DFT calculations to assess its thermodynamic stability and electronic structure. Specifically, we computed the decomposition enthalpy into plausible ternary and binary compounds. To preserve stoichiometry and capture realistic decomposition pathways, we selected only a few specific ternary and binary compounds as likely decomposition products, based on their known stability within the Cs–Cu–Sb–Cl chemical space.

Figure 4a summarizes the decomposition enthalpies for various combinations of products. Several different pathways have negative enthalpy, indicating a high susceptibility of the Cu-based perovskite phase to decompose. The strongest driving force is found for decomposition into the ternary compounds with Cs_3_Sb_2_Cl_9_, confirming its thermodynamic preference over the perovskite phase.

**Figure 4:**
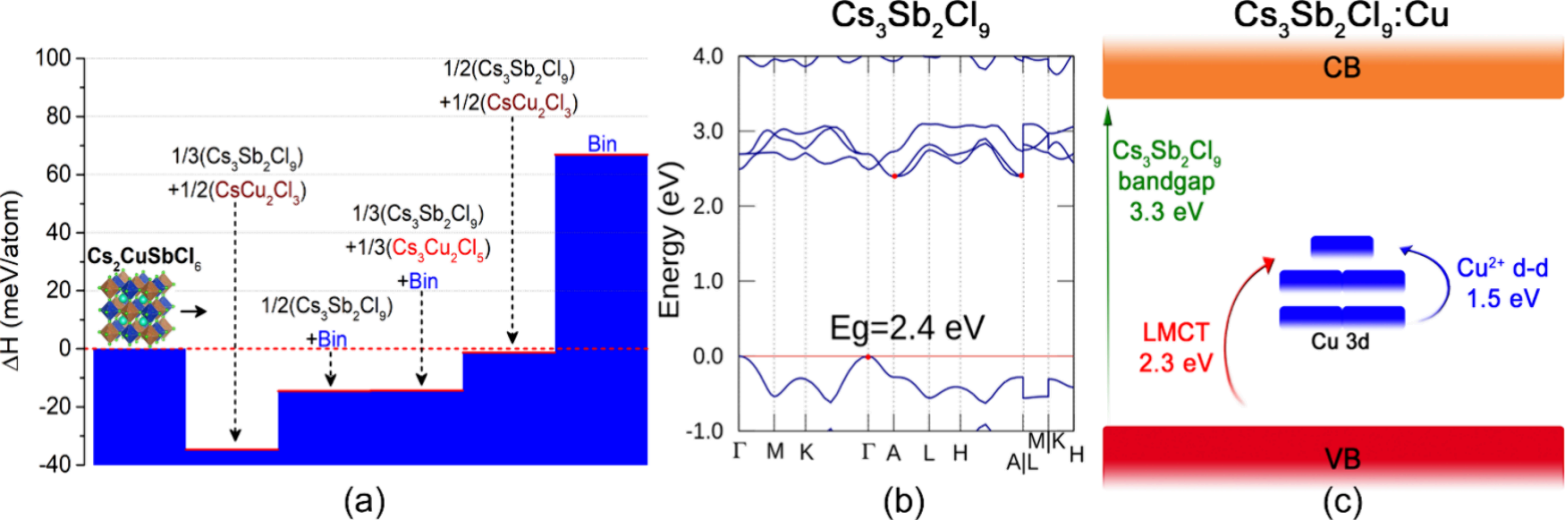
(a) Decomposition enthalpies of Cs_2_CuSbCl_6_ into different ternary and binary chlorides (labeled as Bin). (b) Electronic band structure of trigonal Cs_3_Sb_2_Cl_9_ at the PBE level. (c) Schematic band diagram showing localized Cu 3d states in relation to Cs_3_Sb_2_Cl_9_.

A deeper insight into the chemical origin of such instability comes from the band structure and projected density of states (pDOS) of the Cu HDP compared with its Ag analogue, which on the contrary has been successfully synthesized in several reports and exhibits high thermodynamic stability, evidenced by positive decomposition enthalpies toward ternary and binary compounds (see Figure S28). The pDOS of cubic Cs_2_CuSbCl_6_ and Cs_2_AgSbCl_6_ calculated at the PBE level are shown in Figure S29. The main peaks of the Cu 3d states are located approximately 2 eV higher in energy than those of the Ag 4d states; this generally destabilizes the 6-fold coordinated CuCl_6_ octahedra.^23^ Moreover, a stronger Ag *d -* Cl *p* coupling results from these states being closer in energy. In contrast, the larger separation between the Cu *d* and Cl *p* states reflects both weaker coupling and lower structural stability. These findings are all consistent with the decomposition enthalpy results, confirming the lower stability of the Cu HDP compared to its Ag analogue. Overall, both the lower stability and the lower band gap of Cs_2_CuSbCl_6_ (see Figure S30) can ultimately be attributed to the higher energy of the Cu *d* states.^23^ To obtain accurate band gap values, we performed *GW* calculations on Cs_2_AgSbCl_6,_ which show PBE underestimates the band gap by ∼0.8 eV (Figure S30). Unfortunately, *GW* calculations fail to converge in the presence of Cu due to localized *d* states, which cause strong electronic correlations and sharp variations in the quasiparticle self-energy.

We expect the defective Cs_4_CuSb_4_Cl_18_ structure in Figure 2c to be one of the different possible configurations which average out over the long-range. Through band structure and pDOS simulations, we investigated this structure configuration and compared it with the defect-free structure of Cs_3_Sb_2_Cl_9_. The bands of Cs_3_Sb_2_Cl_9_ show a gap of 2.4 eV (Figure 4b) that underestimated as expected from PBE calculations. The VBM is mainly composed of Cl p and Sb 5s states, while the conduction band minimum (CBM) originates primarily from Sb p and Cl p states (Figure S28). As Cu^2+^ in the 3d^9^ configuration is not fully occupied, this configuration plays a key role and may give rise to magnetization effects and Hubbard corrections necessary (DFT+U). Under these conditions, the simulations are challenging and should be interpreted qualitatively. We first studied the system in the nonmagnetic case, and found Cu 3d-derived doping states just above the Fermi energy (Figure S31), which can be associated with two possible electronic transitions: either from Cl ligand states (i.e., near the Fermi level) or from other Cu 3d electrons (above the Fermi level), as illustrated in the scheme in Figure 4c. When magnetization is included the calculations show agreement with the nonmagnetic results (Figure S31). Some Cu 3d-derived doping states appear in the middle of the band gap, while others states exhibit a minor influence, attributed to the overlap of Cu 3d states with Sb s/p and Cl p states at the VBM, while showing no contribution to the CBM, confirming the presence of two optical absorption peaks in the visible and NIR range. On the other hand, DFT+U correction did not yield physically meaningful results in our system, likely due to the instability of the magnetic configuration induced by the partially filled Cu 3d orbitals, which prevented proper convergence and led to unphysical solutions. The applicability of DFT+U corrections in this system remains an open question and warrants further investigation in future studies.

With a combination of XRD, XANES, EXAFS, and optical spectroscopy, we conclude that Cu-doped Cs_3_Sb_2_Cl_9_ NCs are formed instead of Cs_2_CuSbCl_6_ from one-pot hot injection, confirming DFT predictions of limited stability for the HDP. We propose an overall structure bringing together the evidence from long-range and short-range X-ray techniques, in which Cu^2+^ in trigonal pyramidal coordination sits off-centered in empty Cs^+^ sites. The peculiar broad optical absorption in the visible range, previously attributed to a low bandgap of the double perovskite, is rather due to isolated LMCT and d-d transitions at the Cu^2+^ site in Cs_3_Sb_2_Cl_9_, which is a wide bandgap semiconductor.

## Experimental Section

Experimental details are provided in the Supporting Information.

## Supplementary Material



## Notes

Full experimental details regarding the synthesis, characterization techniques, and XANES and DFT simulations; additional TEM images, optical spectra and XANES spectra; Rietveld refinements; EXAFS analyses at the Cu K-edge; DFT optimized atomic structures, band structures and electronic density of states; survey of Cu–Cl bond lengths from the Cambridge Structural Database (PDF)
