# Supplementary material for: Cu-Doped Cs3Sb2Cl9 Nanocrystals: Revisiting the Low Bandgap of Cs2CuSbCl6 Double Perovskites
Source: ACS Mater Lett. 2025 Oct 10;7(11):3626–33. doi: 10.1021/acsmaterialslett.5c01043 (PMC12588299; doi:10.1021/acsmaterialslett.5c01043)
Supplement: Supplementary file 1 [file tz5c01043_si_001.pdf]

Supporting Information for:

Cu-doped $Cs_3Sb_2Cl_9$ nanocrystals: revisiting the low bandgap of $Cs_2CuSbCl_6$ double perovskites

Simone Virga[1], David F. Macias-Pinilla[1], Nicola Dengo[2], Federica Bertolotti[2], Alessandro Longo[3], Fei He[4], Quinten A. Akkerman[4,*], Francesco Giannici[1,*]

[1] Department of Physics and Chemistry, University of Palermo, viale delle Scienze, 90128 Palermo, Italy

[2] Department of Science and High Technology and Total Scattering Laboratory (To.Sca.Lab), University of Insubria, via Valleggio 11, 22100 Como, Italy.

[3] BM16-FAME Beamline, The European Synchrotron Research Facility, 71 Avenue des Martyrs, 38000, Grenoble, France and ISMN-CNR, UOS Palermo, Via Ugo la Malfa 156, 90143, Palermo, Italy

[4] Chair for Photonics and Optoelectronics, Nano-Institute Munich, Department of Physics, Ludwig-Maximilians-Universität (LMU), Königinstraße 10, 80539 Munich, Germany

* corresponding authors: q.akkerman@lmu.de, francesco.giannici@unipa.it

Methods

Materials. Cesium acetate (Cs(OAc), 97%), copper (II) acetate anhydrous ($Cu(OAc)_2$, 98%), antimony (III) acetate ($Sb(OAc)_3$, 97%), oleic acid (OA, 90%), oleylamine (OLAM, 80-90%), 1-octadecene (ODE, 90%), chlorotrimethylsilane (TMSCl, 97%) were purchased from Thermo Scientific. Toluene was purchased from J.T. Baker. n-hexane (>98.5%) was purchased from Carlo Erba Reagents. All materials were used without further purification.

NCs Synthesis. To prepare $Cs_3Sb_2Cl_9$:Cu NCs, 0.65 mmol Cs(OAc), 0.22 mmol $Cu(OAc)_2$, and 0.5 mmol $Sb(OAc)_3$ are were placed in three-necked round-bottom flasks of 50 mL with OA (2.9 ml), OLAM (0.65 ml) and ODE (10 ml), the mixture is stirred at room temperature for about 10 minutes.[1] The mixture is heated to 105 °C under vacuum for about 1h. The reaction mixture is heated under a nitrogen atmosphere, and TMSCl (0.4 ml) is swiftly injected at 165 °C and immediately cooled to room temperature in an ice-water bath. The reaction mixture is then decanted into a centrifugal tube and centrifuged at 9000 rpm for 10 min. The supernatant is removed. The precipitate is redispersed in 10 mL toluene with sonication and centrifuged at 9000 rpm for 10 min. The supernatant is discarded. The precipitate is redispersed in 10 mL hexane with sonication and centrifuged at 5000 rpm for

10 min. The supernatant obtained has a purple color and is assumed to contain the NCs, while the precipitate is discarded.

The synthesis described above was then also repeated without either $Cu(OAc)_2$ or $Sb(OAc)_3$, respectively, or without both $Cu(OAc)_2$ or $Sb(OAc)_3$, and also with different amounts of $Cu(OAc)_2$. In total, five syntheses were performed with Cu:Sb ratios 0.055:1, 0.11:1, 0.22:1, 0.44:1, 2:1. When not specified otherwise, a Cu:Sb ratio of 0.44:1 was used, and this latter synthesis was repeated two times to check for reproducibility. The different experimental conditions of all syntheses are summarized in **Table S1**.

UV-vis-NIR. Absorption spectra were recorded using a Cary 60 UV-Vis Spectrophotometer (Agilent Technologies) and a LAMBDA 1050+ (PerkinElmer).

Transmission electron microscopy (TEM). TEM images were obtained using a JEOL JEM-1011 microscope operating at 80 keV. TEM samples were prepared by drop-casting NC solutions onto copper TEM grids. Segmentation of the nanoparticles (NPs) in the TEM images was performed using ImageDataExtractor by Yildirim and Cole.[2] For the segmentation, uncertainty threshold was kept at 0.0125, and 50 Monte Carlo samples for Bayesian inference were used. The output projected NPs areas were then converted in the equivalent diameter of the sphere projecting the same area. The resulting distribution was fitted using a lognormal distribution.

XRD. Laboratory X-ray diffraction (XRD) patterns of experimental $Cs_3Sb_2Cl_9$ and $Cs_3Sb_2Cl_9$:Cu - second batch were collected using Cu Kα radiation (λ=1.5418 Å) on a Rigaku Miniflex diffractometer equipped with a DTEX detector operating at 30 kV and 10 mA. A few droplets of each colloidal sample were deposited on the surface of a silicon monocrystal zero-background plate with the aid of a micropipette and dried in air within minutes. The measured angular ranges for all datasets are characterized by a $2\theta_{min}$= 4.8° and a $2\theta_{max}$ = 80°, with a 2θ-step of 0.05°. For the $Cs_3Sb_2Cl_9$:Cu sample, XRD data was collected on a laboratory X-ray scattering setup using Mo Kα radiation (0.71 Å) from a microfocus X-ray source (Xenos) collimated to a beam size of ≈1 × 1 mm. Dispersions of NCs in n-hexane were drop-casted onto adhesive tape (3 m Scotch Magic 810) and measured in transmission mode after drying. The data was collected using a Dectris Pilatus 100K detector. The pattern was transformed to Cu Kα for comparison. The VESTA software[3] was used to simulate the diffraction patterns of trigonal α-$Cs_3Sb_2Cl_9$ (ICSD #22075),[4] orthorhombic β-$Cs_3Sb_2Cl_9$ (ICSD #2066),[5] and Cs_2CuCl_4 (ICSD #15699)[6] reported in Figure 1a of the main text. Starting from these structural models, Rietveld refinements were performed using the

experimental data in the 8.5° ≤ 2θ ≤ 72° range with the TOPAS software.[7] In Table S2 all the refined parameters are reported, together with the corresponding best fits for the different models refined against the data for the samples $Cs_3Sb_2Cl_9$ and $Cs_3Sb_2Cl_9$:Cu (two batches, one collected in transmission and one in Bragg-Brentano geometry). For the latter, a spherical harmonics description was used to describe the (weak) preferred orientation effects found in the experimental data. Given the limitations in laboratory data quality and the complexity of the samples (often exhibiting the co-presence of multiple phases), the atomic coordinates were fixed to the values reported in the corresponding Crystallographic Information Files.

XAFS. X-ray absorption spectra were acquired at the Cl, Cu and Sb K-edges and at the Sb L_3-edge. The Cl K-edge and Sb L_3-edge were acquired at the ID26 beamline of the European Synchrotron Radiation Facility (ESRF, France), by collecting the KL_3 (Cl K-edge) and L_3M_5 emission lines (Sb L_3-edge) in high-energy resolution fluorescence detection mode. The Sb K-edge was acquired in transmission mode at 80 K at the SAMBA beamline of Synchrotron SOLEIL (France). The Cu K-edge spectra of the suspensions were acquired at room temperature in fluorescence mode at the BM23 beamline of the ESRF. For the Cl K-edge and Sb L_3-edge, the suspensions were deposited on paper and cooled at 12 K to minimize radiation damage. For the Sb K-edge, the dry sample was pressed with BN and cooled at 80 K. Data reduction and EXAFS analysis was performed with Viper [8] using theoretical scattering paths generated with FEFF9.[9] Ab initio simulations of the near-edge spectra were performed with FDMNES.[10]

Table S1. Cation amounts and resulting copper-antimony molar ratios for each reaction.

$Cu(OAc)_2$ mmol	$Sb(OAc)_3$ mmol	Cs(OAc) mmol	Cu:Sb Ratio
0.22	0.5	0.65	0.44:1*
0	0.5	0.65	0:1
0	0.5	0	0:1 (Without Cs)
0.22	0	0.65	1:0
0.0275	0.5	0.65	0.055:1
0.055	0.5	0.65	0.11:1
0.11	0.5	0.65	0.22:1
1	0.5	0.65	2:1
* Reference cation ratio from ref. [1].			

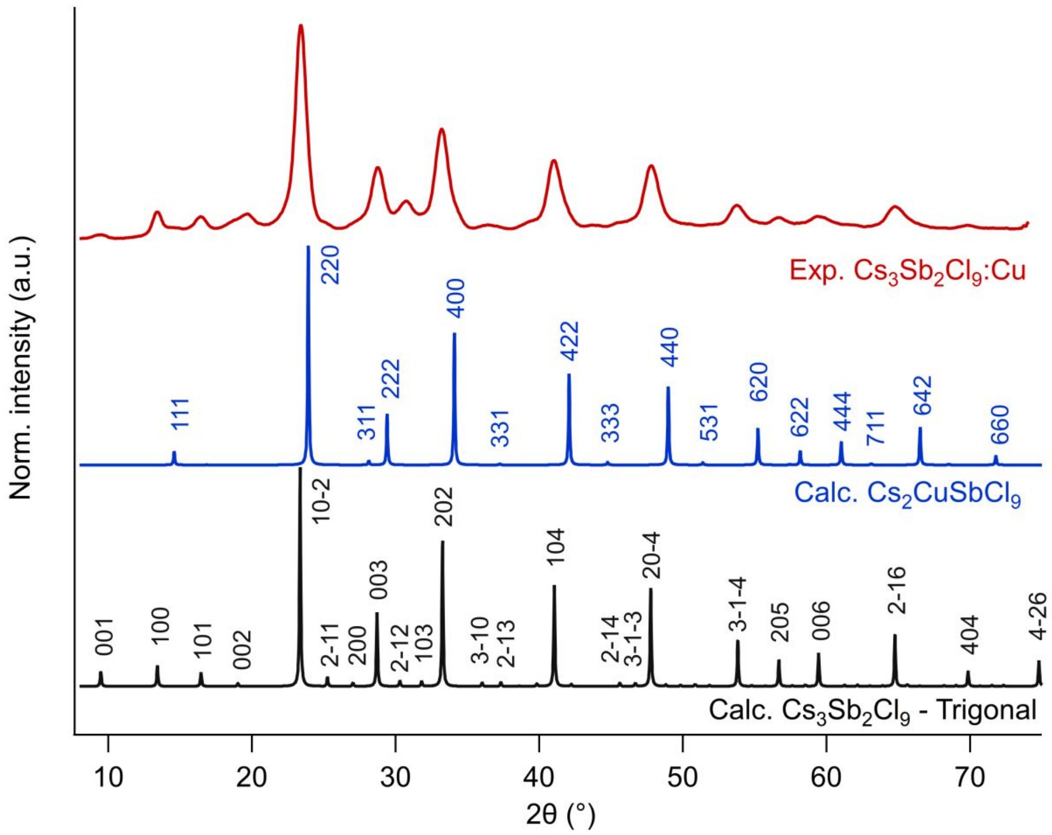

Figure S1. XRD pattern (for λ=1.5418 Å), of the sample $Cs_3Sb_2Cl_9$:Cu compared with simulations of the XRD patterns of trigonal $Cs_3Sb_2Cl_9$ and $Cs_2CuSbCl_6$, together with peak indexing for each phase.

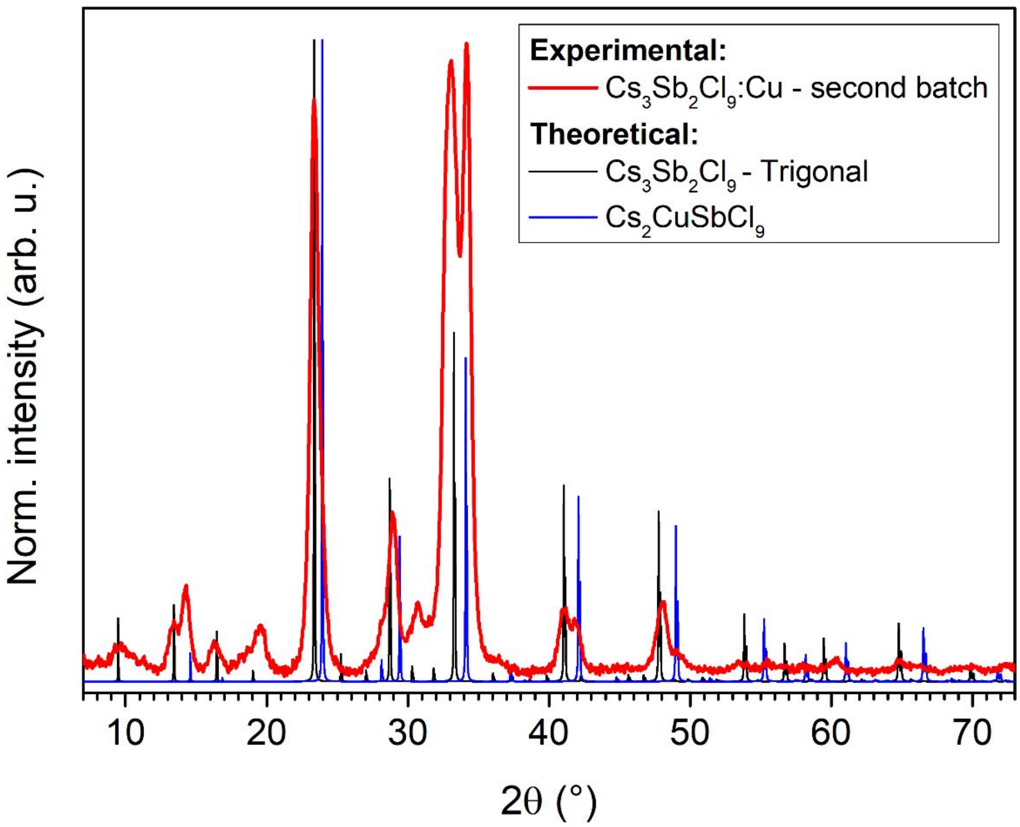

Figure S2. XRD pattern of a second batch of $Cs_3Sb_2Cl_9$:Cu compared with simulations of the XRD patterns of trigonal $Cs_3Sb_2Cl_9$ and $Cs_2CuSbCl_6$.

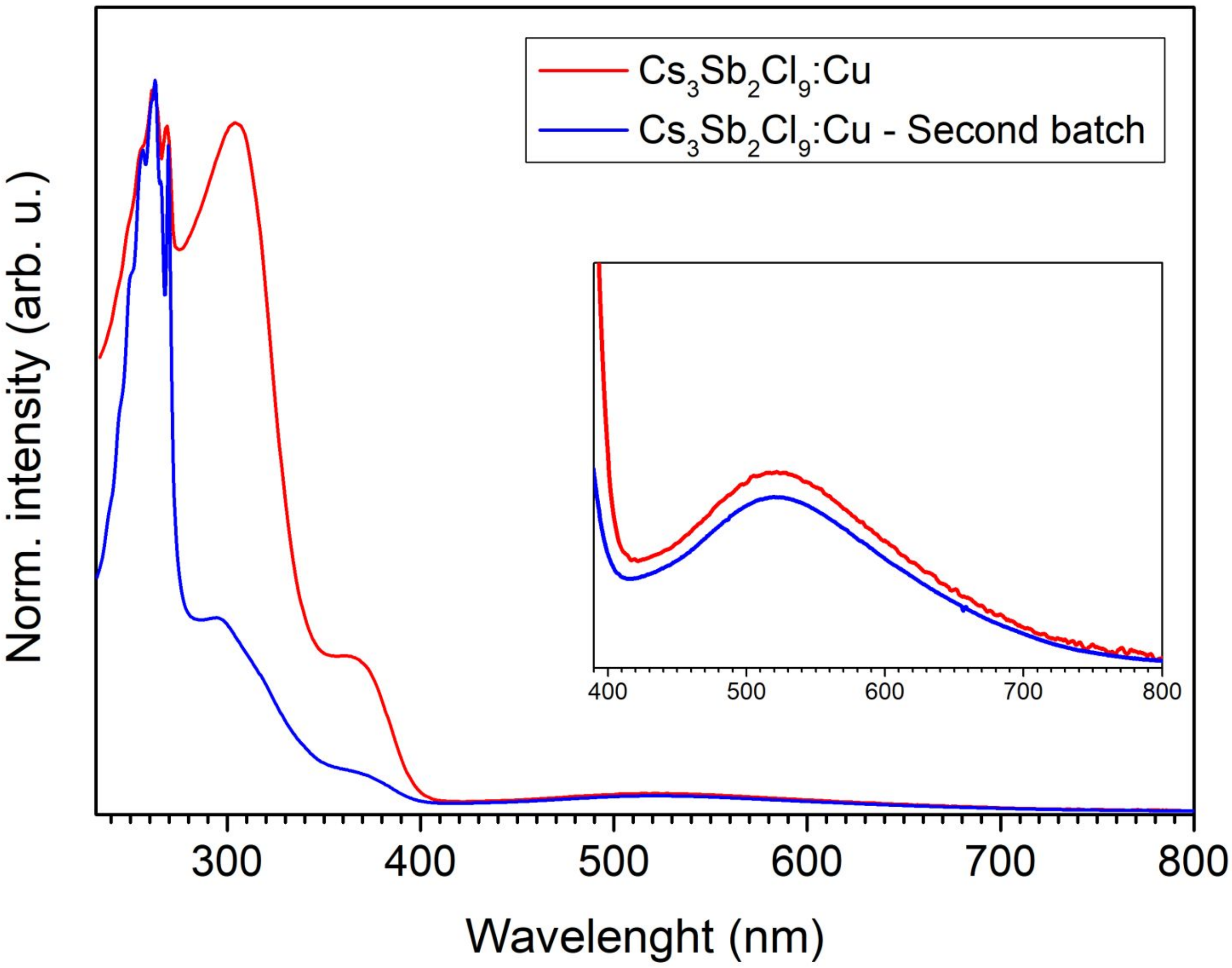

Figure S3. UV-vis absorption spectra of two different batches of $Cs_3Sb_2Cl_9$:Cu synthesis.

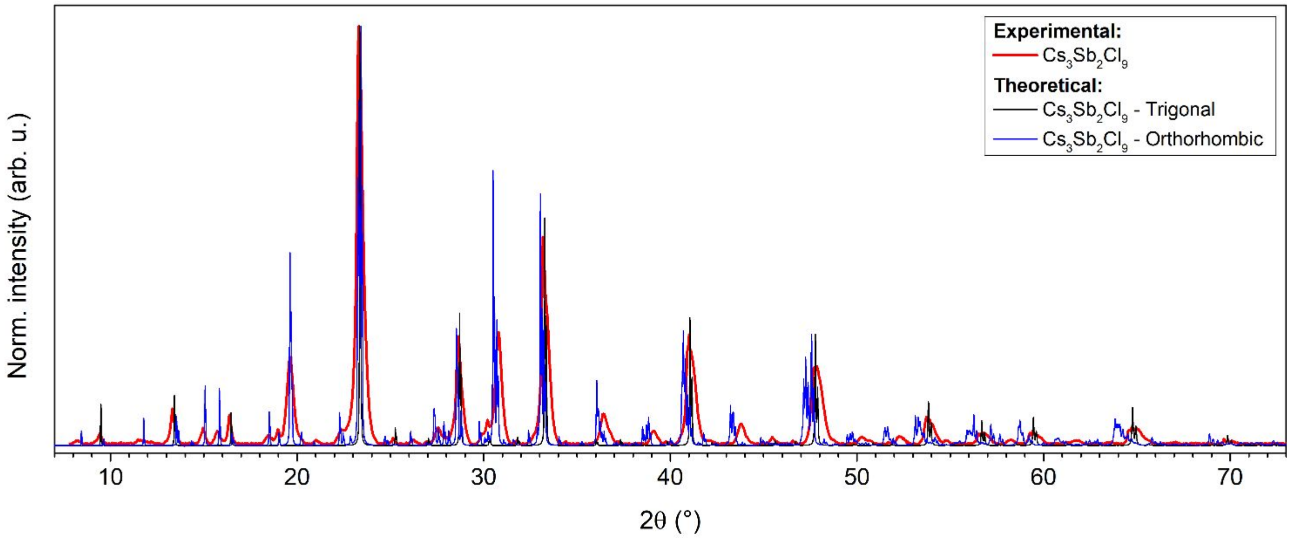

Figure S4. XRD pattern, using Cu Kα radiation (λ=1.5418 Å), of the experimental $Cs_3Sb_2Cl_9$ compared with simulations of the XRD patterns of trigonal and orthorhombic $Cs_3Sb_2Cl_9$.

Rietveld Refinements

Table S2. Lattice parameters of each phase and respective R_p derived from Rietveld refinements.

**Sample**	**Trigonal $Cs_3Sb_2Cl_9$**			**Orthorhombic $Cs_3Sb_2Cl_9$**				**Cubic $Cs_2CuSbCl_6$**		**R_p %**
	a (Å)	c (Å)	%*	a (Å)	b (Å)	c (Å)	%*	a (Å)	%*	
**$Cs_3Sb_2Cl_9$**	7.620(3)	9.276(7)								43.47
				18.647(7)	7.543(2)	3.192(6)				22.84
	7.620(2)	9.318(5)	33.00(2)	18.620(4)	7.593(1)	3.028(1)	67.00(2)			14.56
**$Cs_3Sb_2Cl_9$:Cu**	7.630(9)	9.33(2)								16.48
								10.785(1)		20.94
**$Cs_3Sb_2Cl_9$:Cu - Second batch**	7.56(2)	9.25(4)								33.32
								10.672(8)		30.63
	7.681(5)	9.196(6)	82.00(3)					10.478(5)	18.00(3)	19.96

* Contribution of each crystalline phase in the mixed-phase Rietveld refinement.

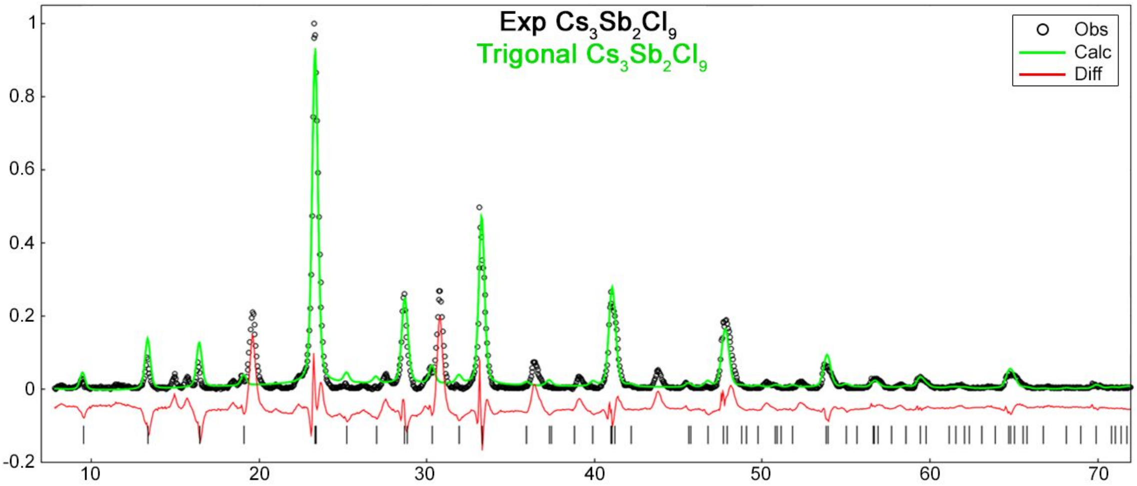

Figure S5. Rietveld refinement of the experimental $Cs_3Sb_2Cl_9$ performed with trigonal $Cs_3Sb_2Cl_9$.

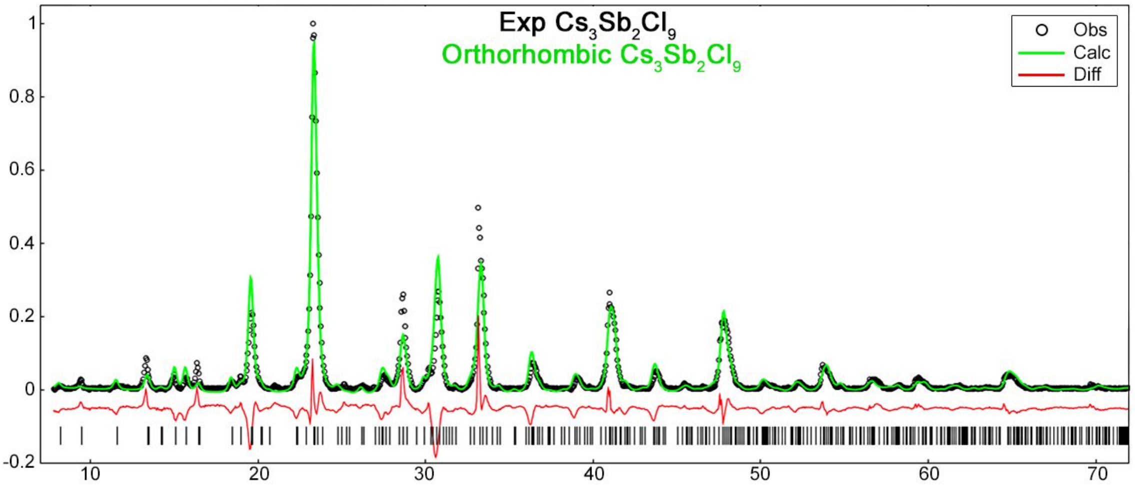

Figure S6. Rietveld refinement of the experimental $Cs_3Sb_2Cl_9$ performed with orthorhombic $Cs_3Sb_2Cl_9$.

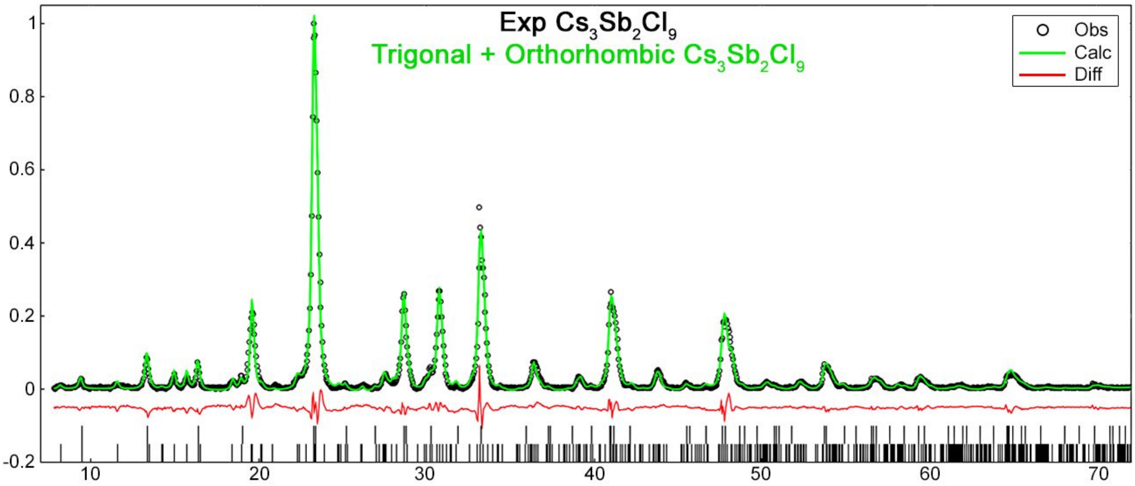

Figure S7. Rietveld refinement of the experimental $Cs_3Sb_2Cl_9$ performed with the two phases, trigonal and orthorhombic, of $Cs_3Sb_2Cl_9$.

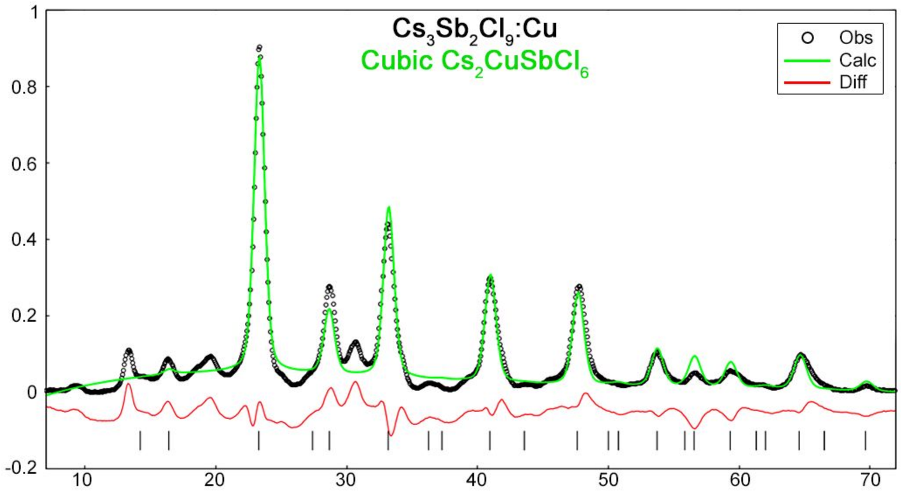

Figure S8. Rietveld refinement of the sample $Cs_3Sb_2Cl_9$:Cu performed with cubic $Cs_2CuSbCl_6$.

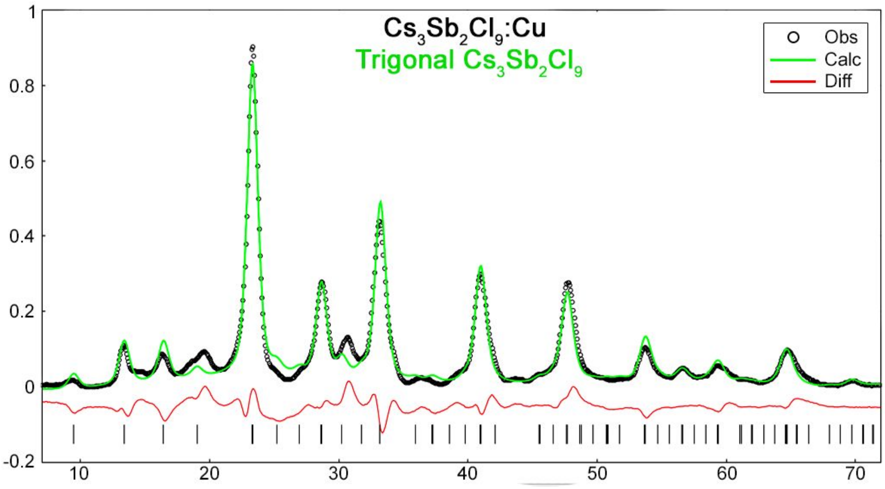

Figure S9. Rietveld refinement of the sample $Cs_3Sb_2Cl_9$:Cu performed with trigonal $Cs_3Sb_2Cl_9$.

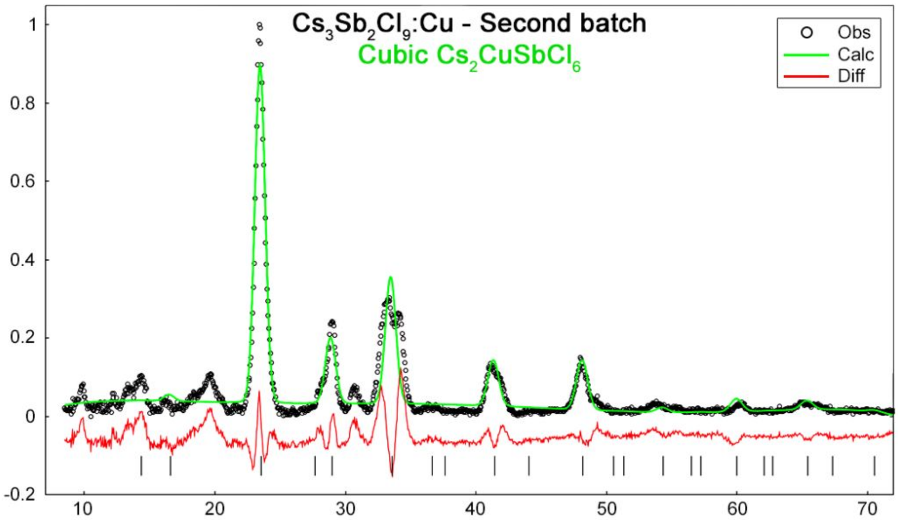

Figure S10. Rietveld refinement of the second batch of the sample $Cs_3Sb_2Cl_9$:Cu performed with cubic $Cs_2CuSbCl_6$.

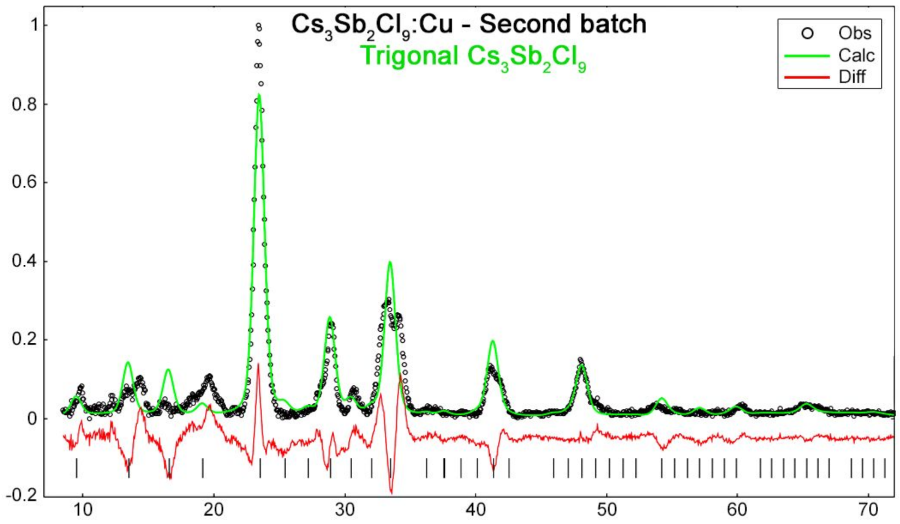

Figure S11. Rietveld refinement of the second batch of the sample $Cs_3Sb_2Cl_9$:Cu performed with trigonal $Cs_3Sb_2Cl_9$.

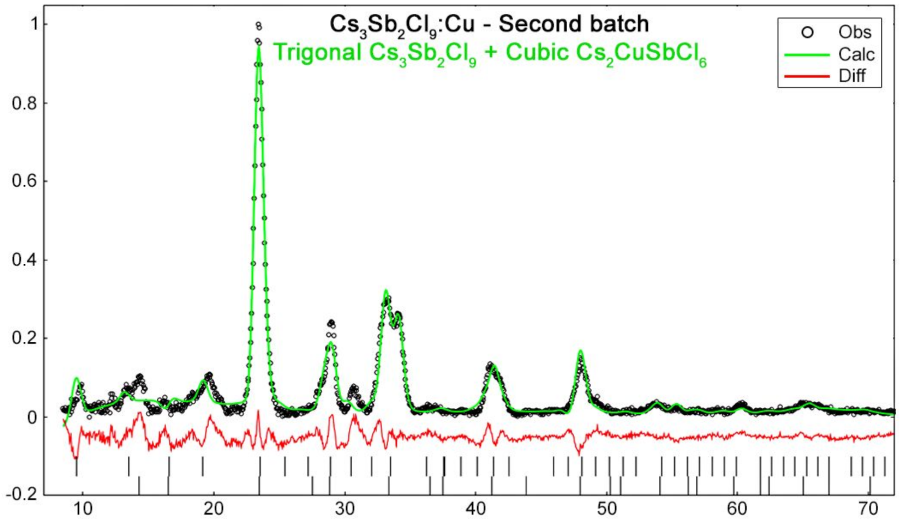

Figure S12. Rietveld refinement of the second batch of the sample $Cs_3Sb_2Cl_9$:Cu performed with trigonal $Cs_3Sb_2Cl_9$ and cubic $Cs_2CuSbCl_6$.

Cu-Cl bond length distributions

The Cu-Cl bond lengths from the Cambridge Structural Database follow a slightly skewed distribution which peaks at 2.25 Å: this is not a bimodal curve that could be used to discriminate between Cu^{+}-Cl and Cu^{2+}-Cl. In particular, the Cu^{+}-Cl bond length distribution also peaks at 2.25 Å, while such a histogram is unreliable for Cu^{2+}-Cl because very few data sources specify the +2 oxidation state, while confirming the 2.25 Å average.

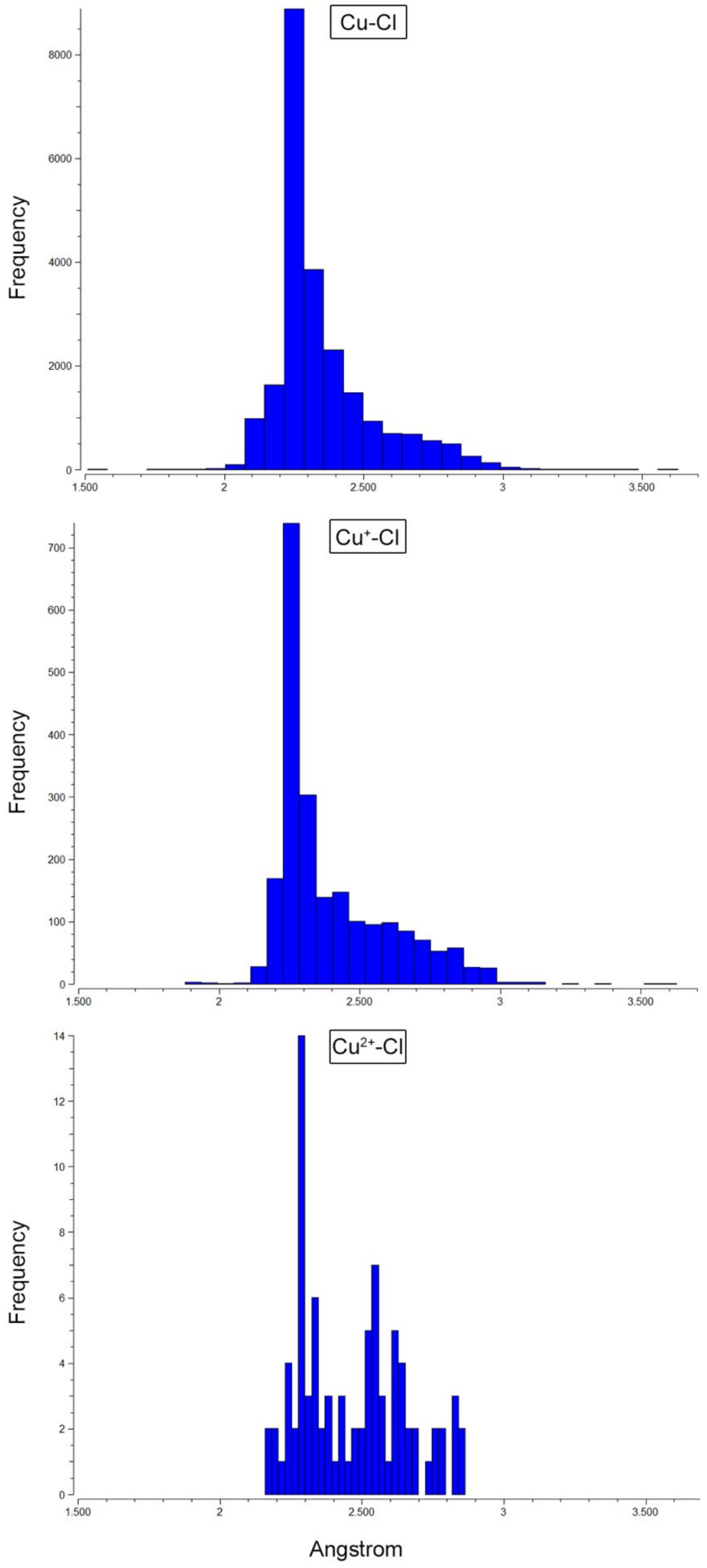

Figure S13. Cu-Cl bond length distribution from the Cambridge Structural Database. Top: Cu-Cl for all oxidation states; middle: Cu^{+}-Cl; bottom: Cu^{2+}-Cl. The latter plot is marred by the fact most data entries do not specify copper in the +2 oxidation state.

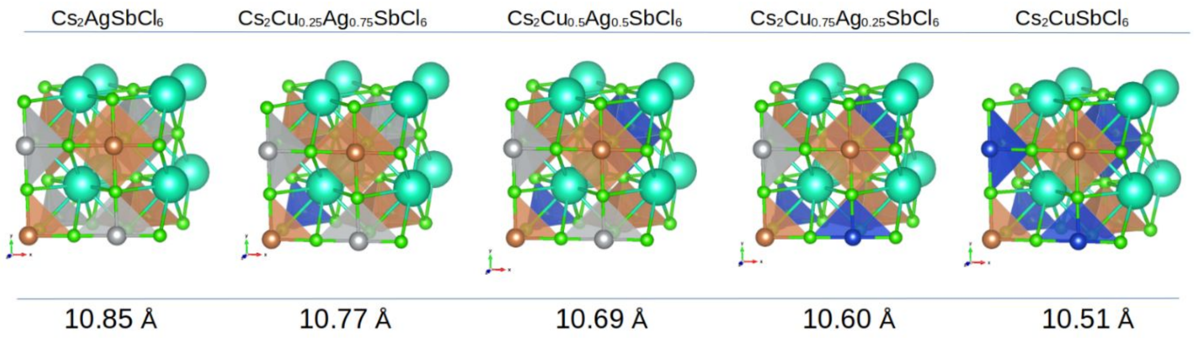

Figure S14. Optimized DFT lattice parameters of cubic mixed compositions of $Cs_2AgSbCl_6$ and $Cs_2CuSbCl_6$.

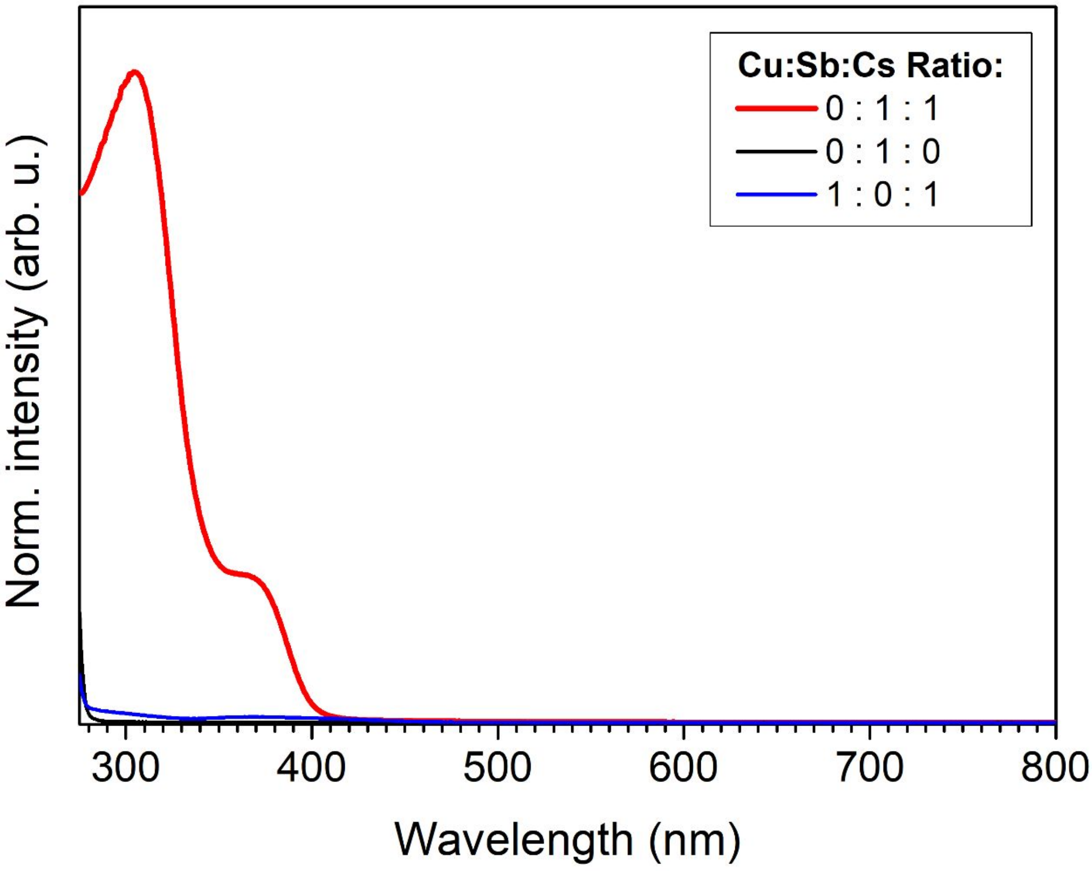

Figure S15. UV-vis absorption spectra of reactions carried out in the absence of either Cu (red line) or Sb (blue line), and in the absence of Cu and Cs (black line).

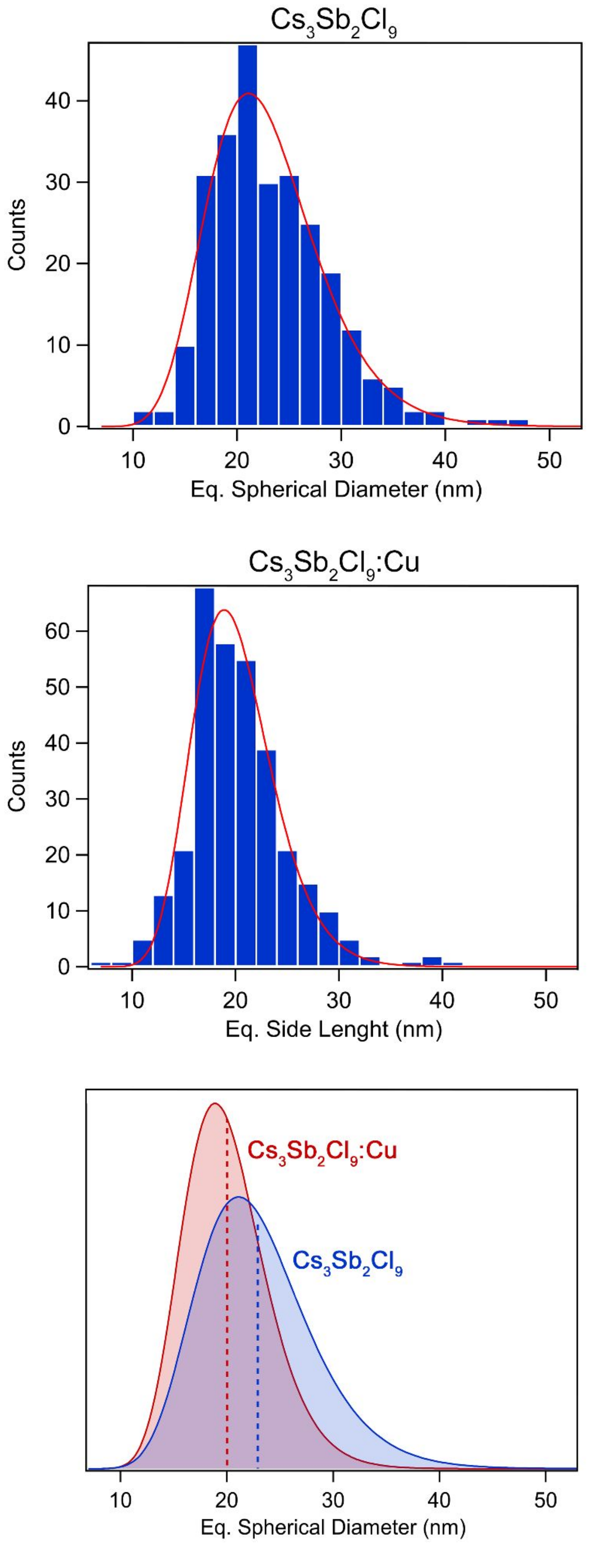

Figure S16. Nanoparticles distribution, fitted using a lognormal distribution, of the two samples $Cs_3Sb_2Cl_9$ and $Cs_3Sb_2Cl_9$:Cu and their comparison.

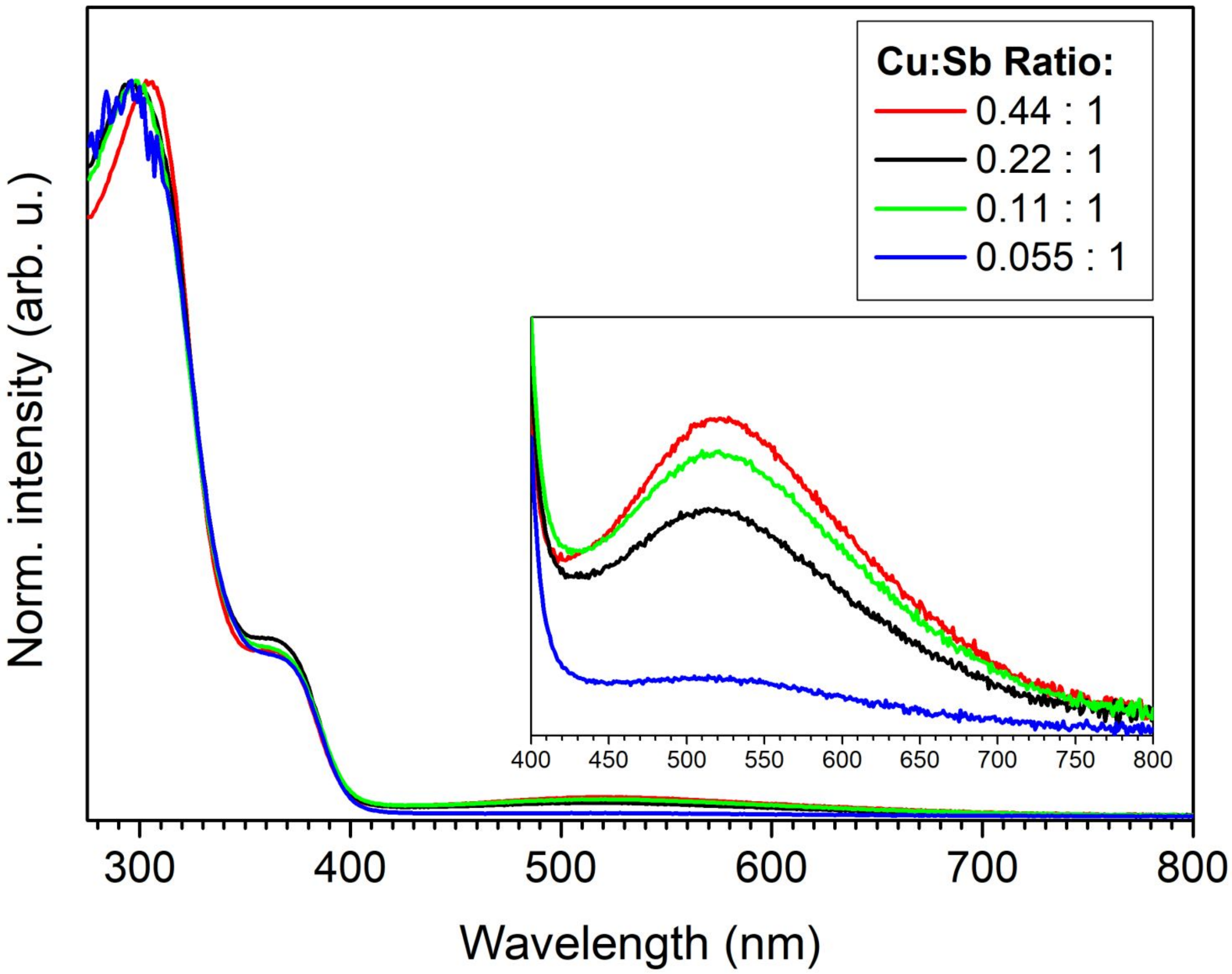

Figure S17. UV-vis absorption spectra of reactions with lower Cu:Sb ratios compared to the reference reaction.

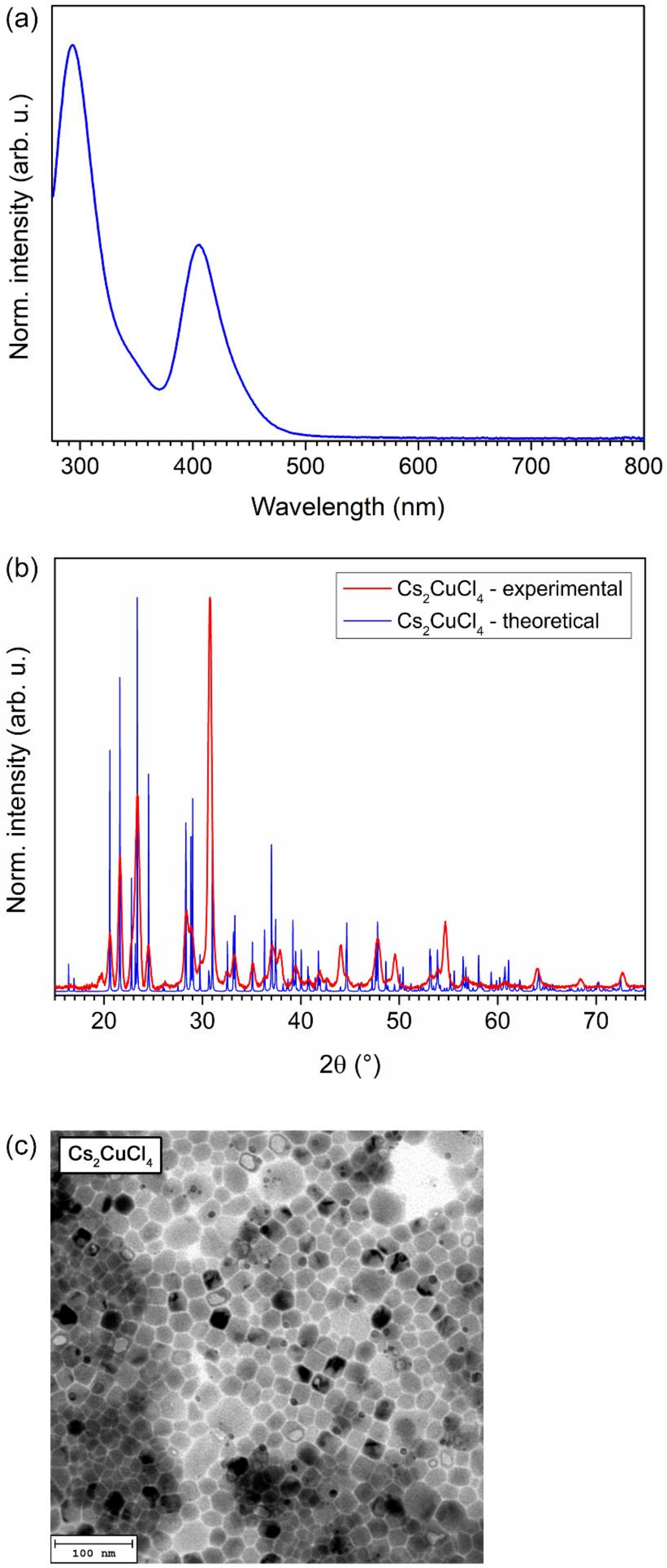

Figure S18. (a) UV-vis absorption spectrum of Cs_2CuCl_4. (b) XRD pattern of the experimental Cs_2CuCl_4 compared with simulation of the XRD patterns of Cs_2CuCl_4. (c) TEM images of Cs_2CuCl_4.

EXAFS

The EXAFS analysis was performed with Viper using theoretical Cu-Cl scattering paths generated with FEFF9 from the Cs_2CuCl_4 cluster. The parameters obtained are shown in the following table.

Table S3. Local structure parameters determined from X-ray absorption spectroscopy (EXAFS). N, R and σ^2 are coordination number, interatomic distance and Debye-Waller factor, respectively. Fittings were performed in R space from about 0.5 to 2.9 Å. Estimated uncertainty is reported in parentheses.

Sample	N	R (Å)	σ^2 (Å^2)	R-factor (%)
Cs_2CuCl_4	4	2.250(5)	0.008(1)	18.55
$Cs_3Sb_2Cl_9$:Cu	3	2.289(5)	0.007(1)	12.84

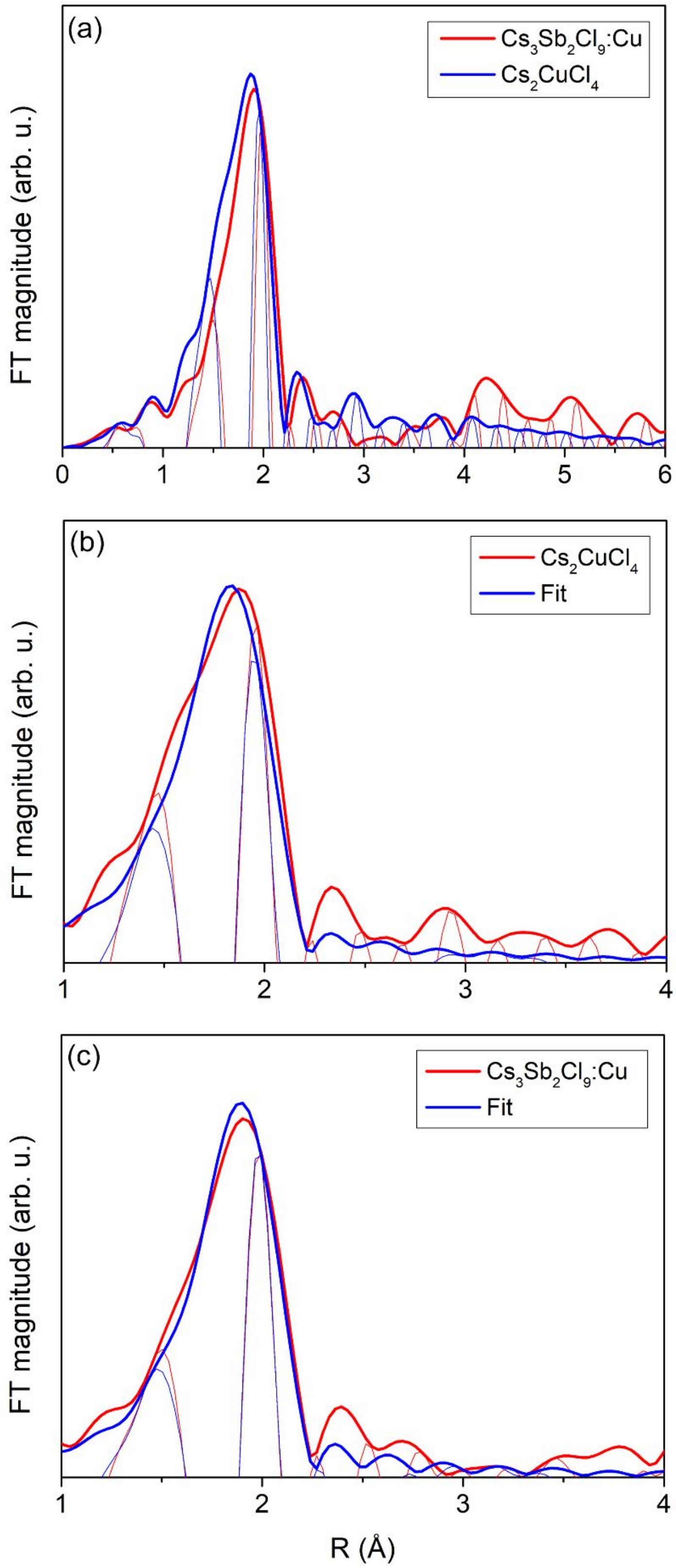

Figure S19. (a) Experimental Fourier-transformed EXAFS spectra (red) of $Cs_3Sb_2Cl_9$:Cu and Cs_2CuCl_4 at the Cu K-edge and best fit (blue) for (b) Cs_2CuCl_4 and (c) $Cs_3Sb_2Cl_9$:Cu. The modulus and imaginary phases are shown.

XANES simulations

XANES simulations on the crystal structures were carried out with the finite difference methods (FDM) with the FDMNES software. The XANES spectra of the $CuCl_n$ isolated clusters were simulated with the FDM approach, with a R_{max} of 4 Å, fixing the vacuum potential parameter V_{max} at -6.0 eV to account for the potential outside the cluster.

For the trigonal pyramid clusters $CuCl_3$ (with α = 102°), different semiempirical screening parameter values were subsequently employed: this accounts for the partial compensation of the core-hole potential by surrounding electrons. Upon photoexcitation, the creation of a core-hole on the absorbing atom induces a strong perturbation in the local electronic potential. However, this core-hole does not act as a fully +1 charge towards the photoelectron, since other electrons and neighboring atoms partially screen its effect. The semiempirical screening parameter ranges from 0 to 1: these two limiting values correspond to either no screening, where the full impact of the unscreened core-hole is included in the simulation, or complete screening, where the core-hole potential is fully neutralized. The effect of the screening parameter on the simulations is shown in **Fig. S23**. An intermediate screening value of 0.5 provided the best agreement with experimental XANES spectra for the trigonal pyramidal cluster $CuCl_3$. This is consistent with a situation where partial screening arises from electron density of the surrounding Cl^- ligands.

The XANES spectra of the extended solids were simulated with periodic boundary conditions using the Green function approach with muffin tin potentials, where the SCF function and the R_{max} parameter were increased until convergence, achieved around 8-9 Å.

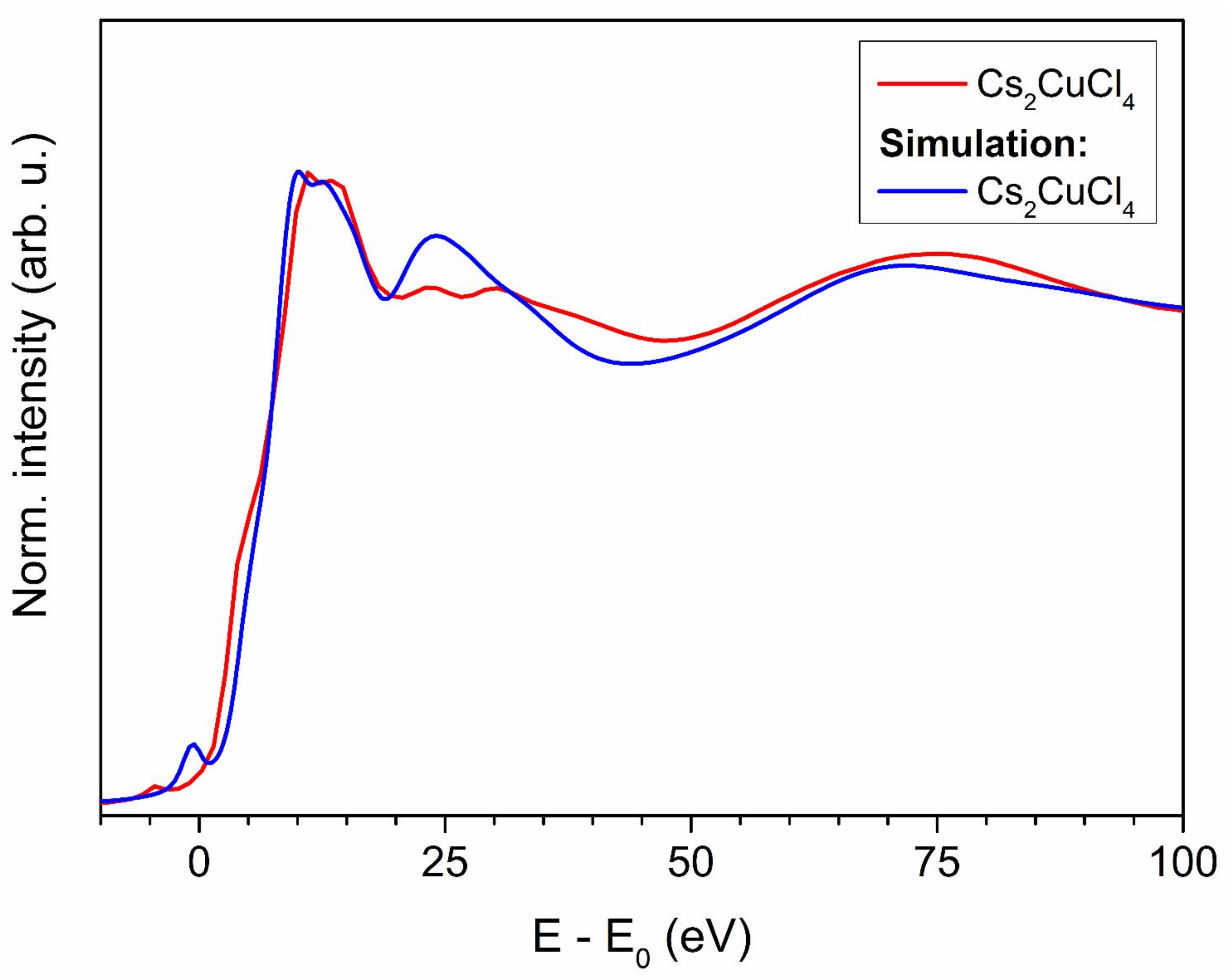

Figure S20. XANES spectra for the Cu K-edge of experimental Cs_2CuCl_4 compared with the XANES simulation of Cs_2CuCl_4.

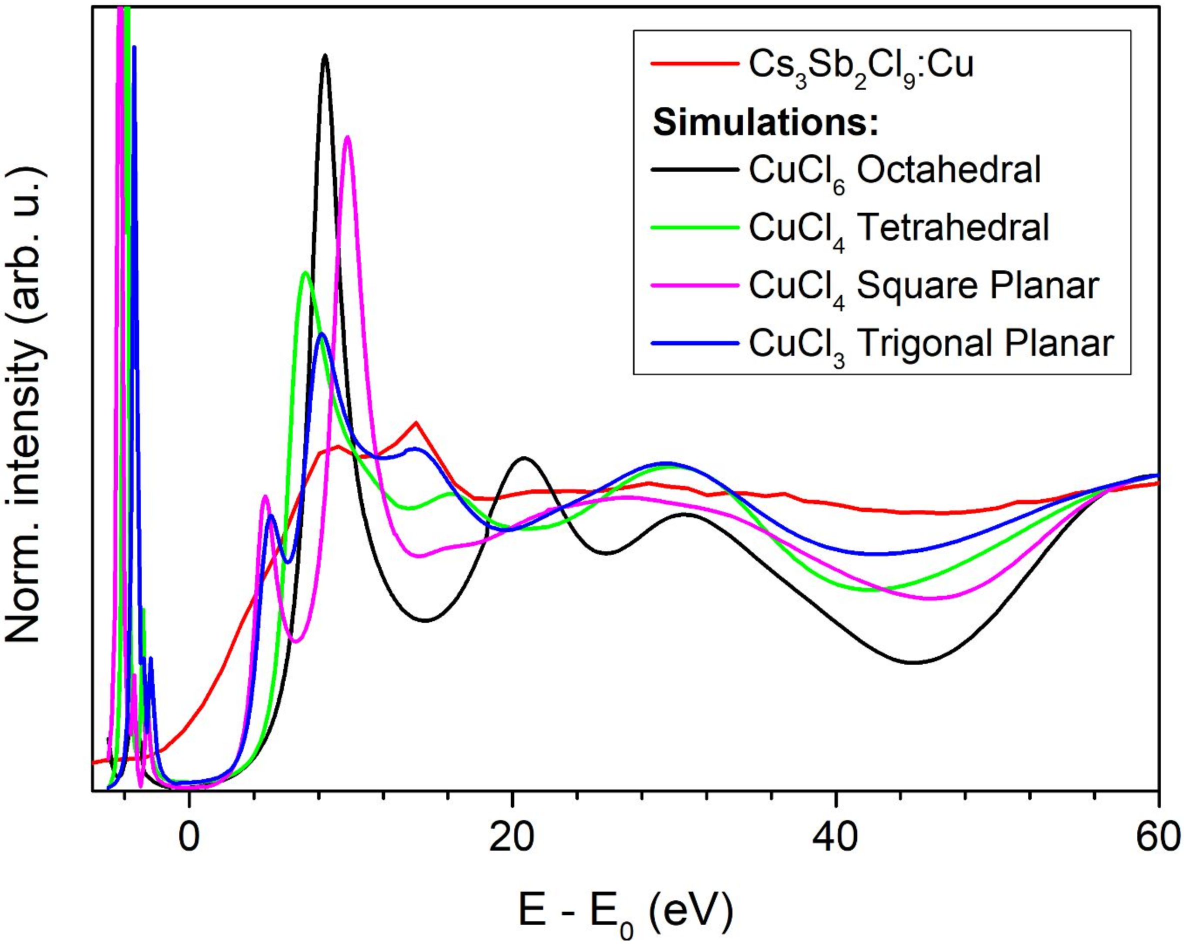

Figure S21. Cu K-edge XANES spectrum of $Cs_3Sb_2Cl_9$:Cu (red) compared with the (non-convoluted) XANES simulations of several $CuCl_n$ complexes.

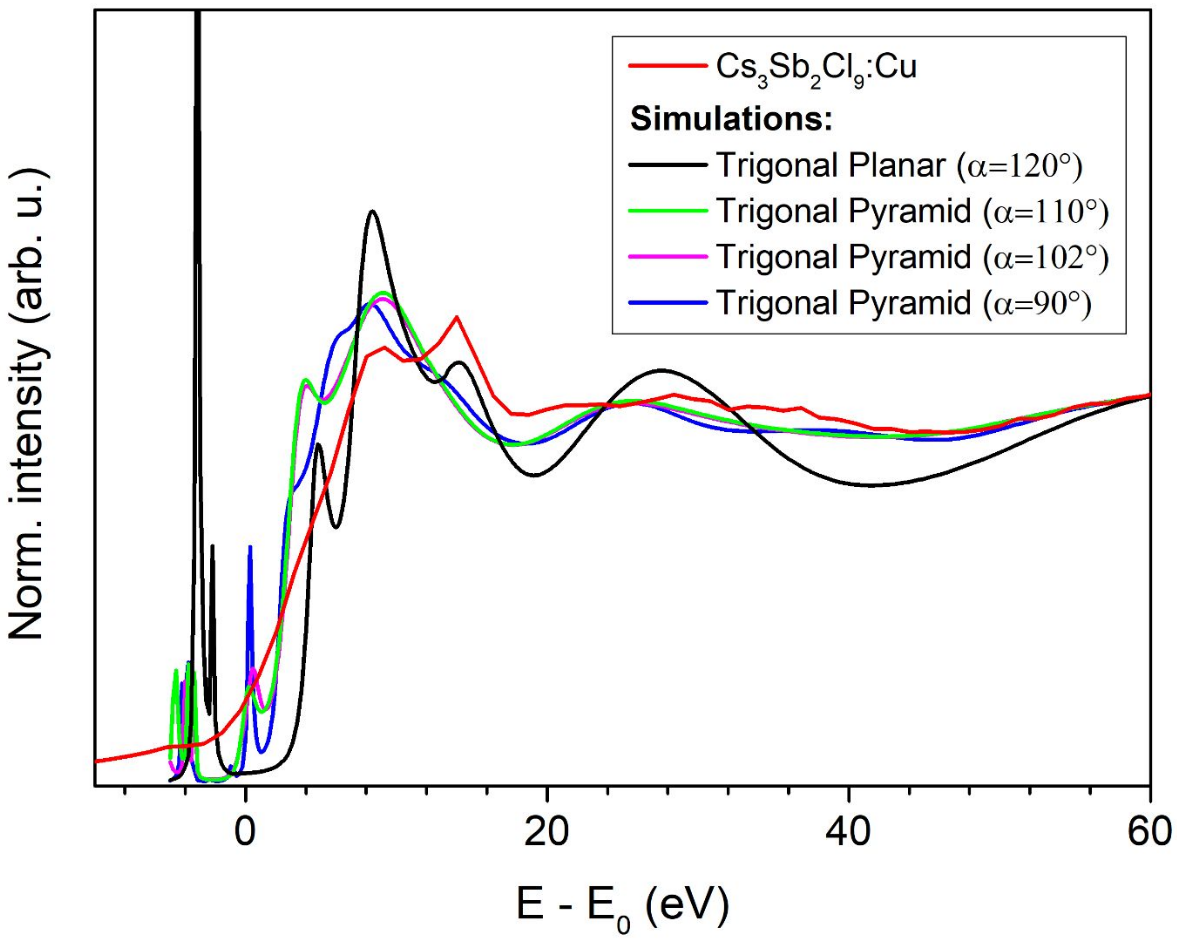

Figure S22. Experimental Cu K-edge XANES spectrum of $Cs_3Sb_2Cl_9$:Cu (red) and XANES simulations (non-convoluted) of a trigonal $CuCl_3$ cluster with different Cl-Cu-Cl angles (α).

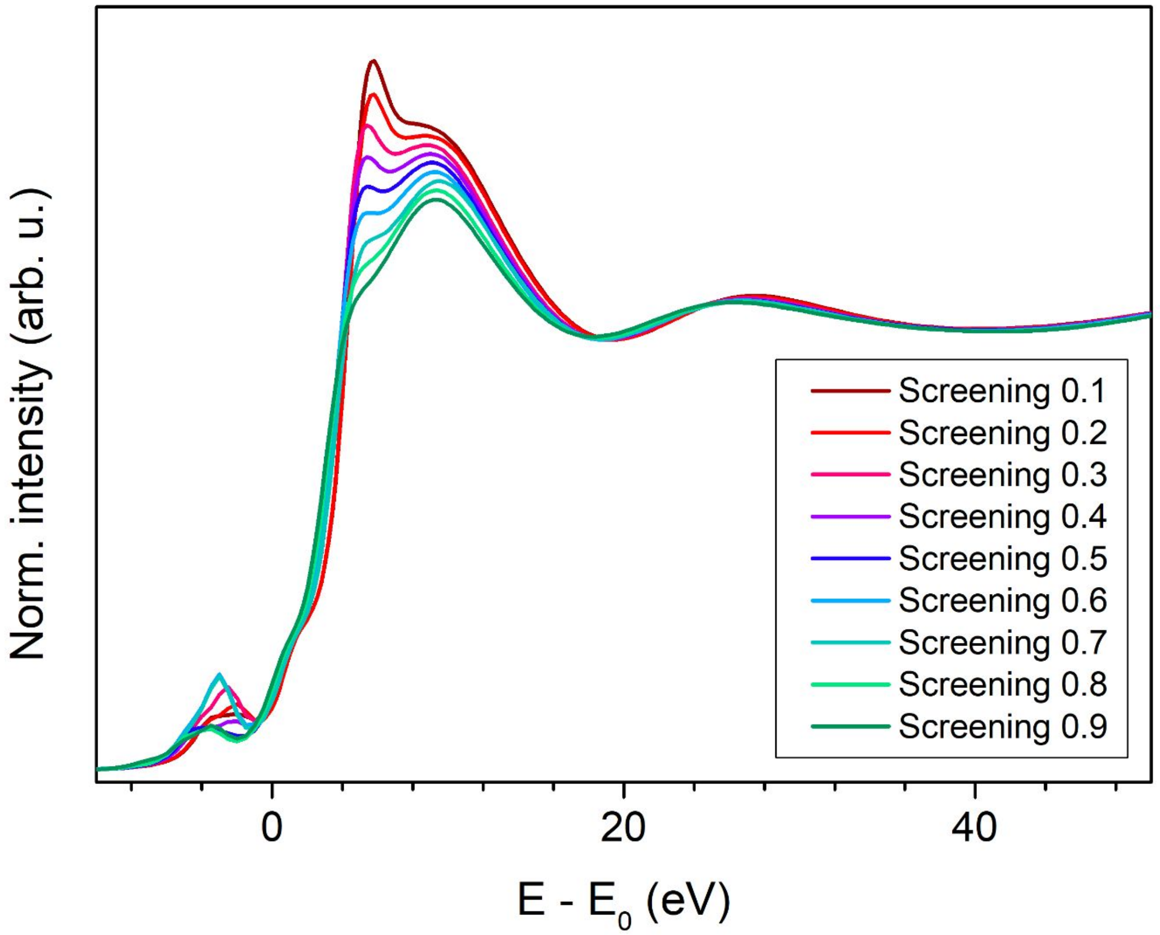

Figure S23. XANES simulations of a trigonal pyramidal $CuCl_3$ cluster (α = 102°) with different values of the semi-empirical *screening* parameter of FDMNES.

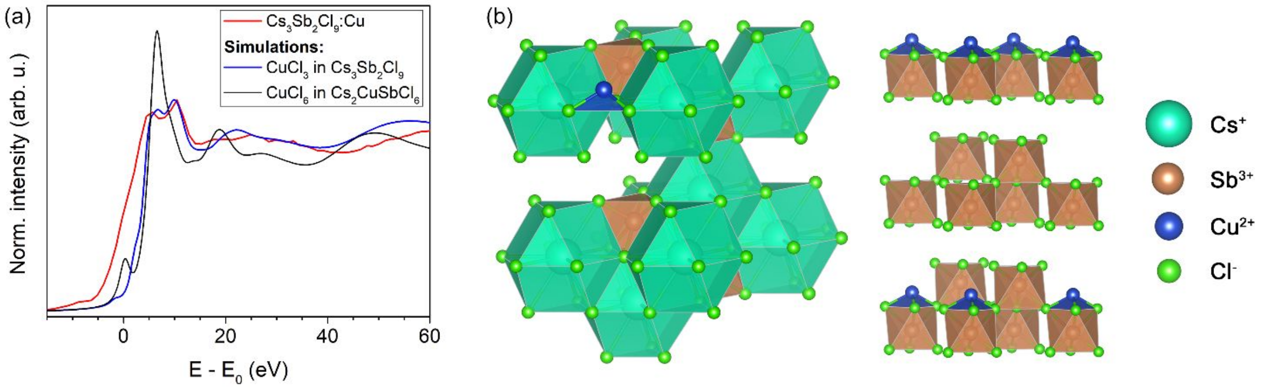

Figure S24. (a) XANES simulations of trigonal pyramidal $CuCl_3$ in $Cs_3Sb_2Cl_9$ and octahedral $CuCl_6$ in $Cs_2CuSbCl_6$. (b) Possible location of a trigonal pyramidal $[CuCl_3]^-$ center, located within the antimony layer of the $Cs_3Sb_2Cl_9$ lattice.

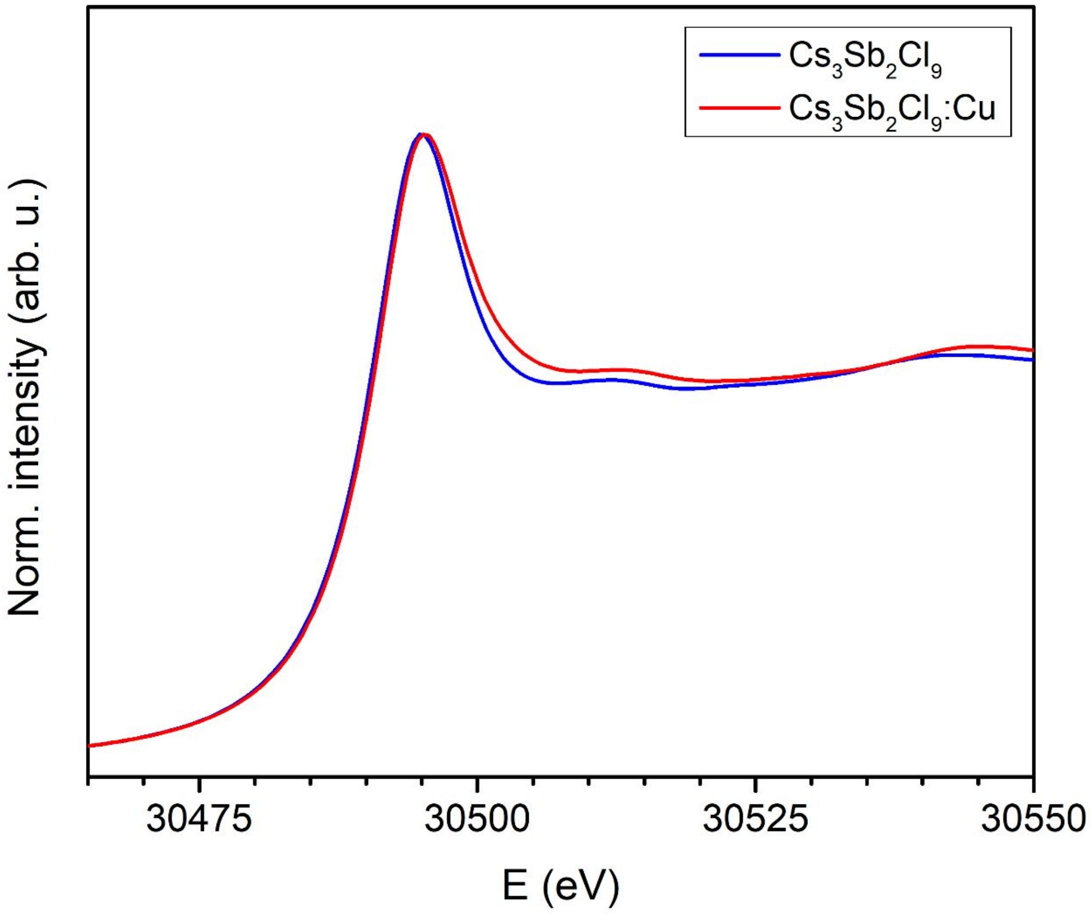

Figure S25. Experimental XANES spectra of $Cs_3Sb_2Cl_9$ and $Cs_3Sb_2Cl_9$:Cu at the Sb K-edge.

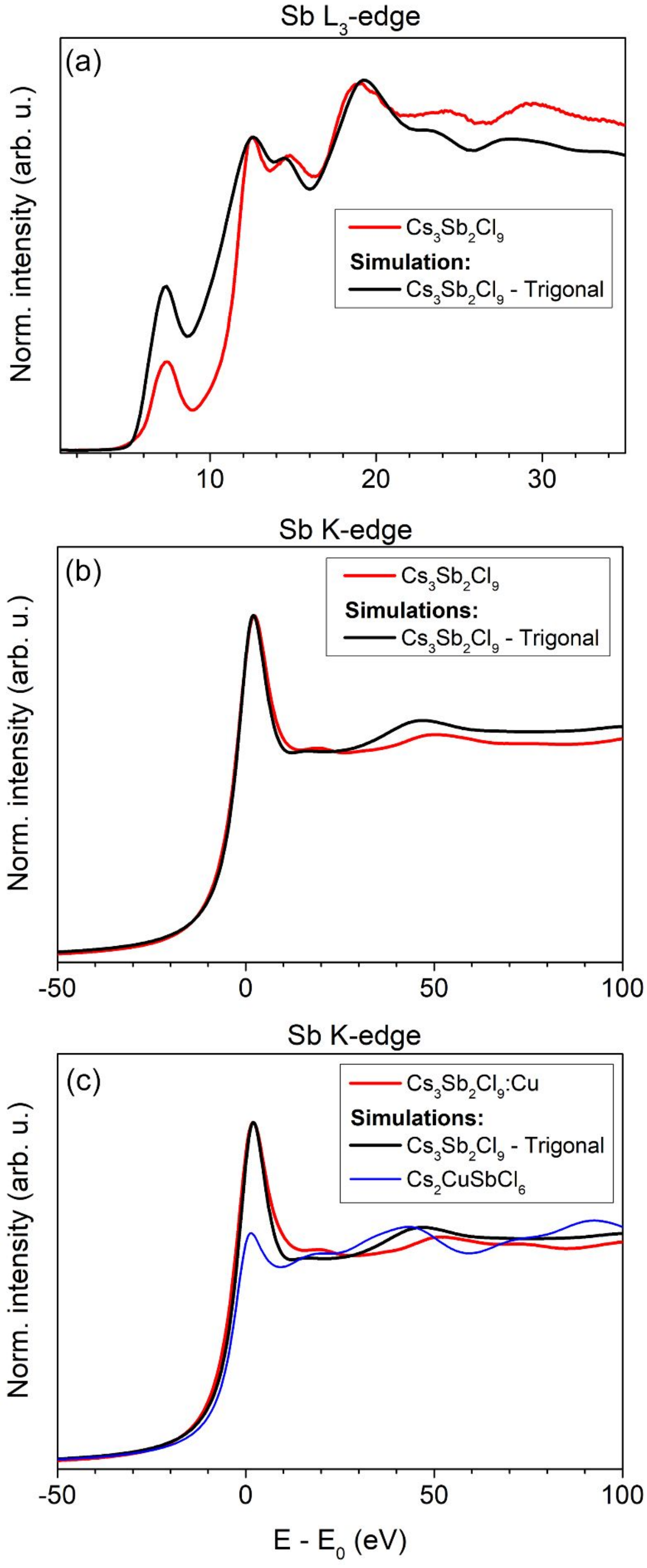

Figure S26. Comparison of experimental XANES spectra (red in all panels) with the simulated spectra of trigonal $Cs_3Sb_2Cl_9$ (black in all panels) and $Cs_2CuSbCl_6$ (blue in all panels). a) Sb L_3-edge of experimental $Cs_3Sb_2Cl_9$ compared with a simulation of trigonal $Cs_3Sb_2Cl_9$. b) Sb K-edge of experimental $Cs_3Sb_2Cl_9$ compared with a simulation of trigonal $Cs_3Sb_2Cl_9$. c) Sb K-edge of experimental $Cs_3Sb_2Cl_9$:Cu compared with simulations of trigonal $Cs_3Sb_2Cl_9$ (black) and $Cs_2CuSbCl_6$ (blue).

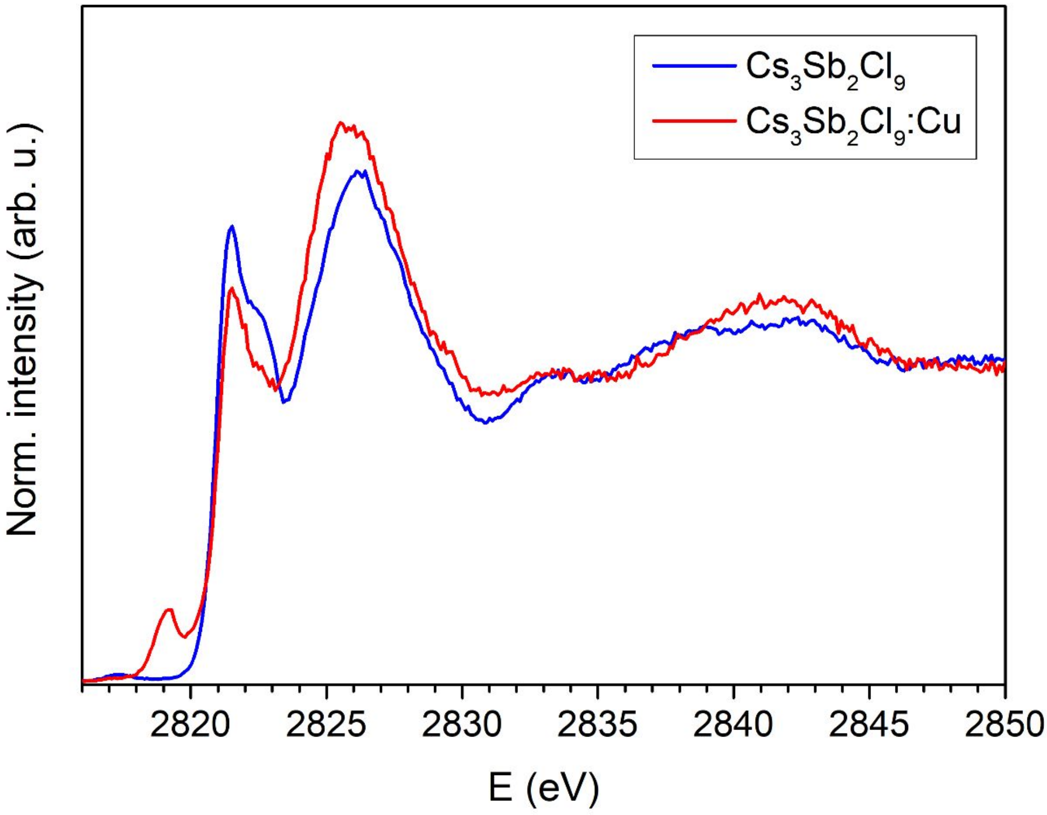

Figure S27. Experimental XANES spectra of $Cs_3Sb_2Cl_9$ and $Cs_3Sb_2Cl_9$:Cu at the Cl K-edge.

Computational Methods

DFT calculations were carried out using the Quantum ESPRESSO (QE) software package[11–13], employing the projector augmented wave (PAW) method[14] and the Perdew-Burke-Ernzerhof (PBE) exchange-correlation functional within the generalized gradient approximation (GGA).[15] The structural optimizations were performed with convergence criteria set to reduce the forces on individual atoms below 0.001 Ry/a_0, where a_0 denotes the Bohr radius and Ry the Rydberg constant. The Brillouin zone was sampled using a Γ-centered Monkhorst-Pack grid chosen to achieve a k-point spacing of approximately 0.15 $Å^{-1}$. Band structures were calculated using QE with the PBE functional. Based on the fully optimized structures, quasiparticle corrections were subsequently obtained within the GW approximation using the Yambo code.[16,17] Scalar-relativistic PAW pseudopotentials from the PSLibrary[18] were employed in combination with the PBE exchange-correlation functional.[19] The kinetic energy cutoff for the wavefunctions was set to 100 Ry, while the charge density cutoff was set as 400 Ry. These cutoff parameters were tested for convergence individually for each structure. The self-consistent field (SCF) calculations were performed with a convergence threshold of 1.0×10^{-6} Ry.

Decomposition Enthalpy

The PBE functional, because of its favorable balance between accuracy and computational cost, is widely adopted for evaluating thermodynamic stability.[20,21] In this work, total energies (E_T) of Ag- and Cu-based halide double perovskites (HDPs) were calculated using PBE, and their decomposition enthalpies (ΔH) were determined as the energy difference between the HDP and its most probable decomposition products:

$$\Delta H = E_T[products] - E_T[HDP]$$

Negative values of ΔH indicate thermodynamic instability with respect to decomposition, whereas positive values suggest inherent stability. The magnitude of ΔH reflects the degree of stability or instability, with large negative values indicating high decomposition propensity and large positive values indicating robust thermodynamic stability. Given the multicationic nature of HDPs, decomposition pathways involving binary and ternary phases were considered for a comprehensive stability assessment.

An analysis of decomposition enthalpies for $Cs_2AgSbCl_6$ is presented in **Fig. S28**. Ag-based perovskites exhibit positive enthalpies with respect to decomposition into binary or ternary products, consistent with significant thermodynamic stability. The ternary compounds analyzed were $Cs_3Sb_2Cl_9$ (*P–3m1*), $CsAgCl_2$ (*P4/nmm1*), and Cs_2AgCl_3 (*Pnma*), along with the binary compounds CsCl, $SbCl_3$, and AgCl. Conversely, Cu-based perovskites were analyzed in relation to the ternary compounds $Cs_3Sb_2Cl_9$ (*P–3m1*), $CsCu_2Cl_3$ (*Cmcm*), $Cs_3Cu_2Cl_3$ (*Pnma*) and binary CsCl, CuCl, $SbCl_3$. Most of the decomposition pathways exhibit negative ΔH values, particularly toward $Cs_3Sb_2Cl_9$, indicating a stronger thermodynamic driving force for decomposition (see **Fig. 4a** in the main text).

Electronic Structure Calculations

The electronic band structure and projected density of states were computed using the PBE functional, which, despite its well-known underestimation of band gaps, reliably describes band dispersion and electronic states. To obtain more accurate band gap estimates for pristine $Cs_2AgSbCl_6$, many-body perturbation theory within the GW approximation was applied, as implemented in the Yambo code.[17] In contrast, GW calculations failed to converge for all Cu-containing phases due to the presence of localized Cu *d* states, which

induce strong electronic correlations and sharp variations in the quasiparticle self-energy.[22–24]

Fig. S30 shows the GW and PBE band structures of $Cs_2AgSbCl_6$, along with the PBE band structures of $Cs_2CuSbCl_6$, $Cs_3Sb_2Cl_9$, and $Cs_3Sb_2Cl_9$:Cu (nominal composition $Cs_4CuSb_4Cl_{18}$). It is well established that GGA functionals (like PBE) underestimate the bandgap of all semiconductors and insulators significantly. We chose not to apply ad hoc corrections like hybrid functionals, as they necessarily add a certain arbitrariness in the degree of Hartree-Fock exchange used. PBE, on the other hand, has proved reliable on structure and thermodynamics, and it is nonetheless useful to derive chemical trends even if bandgaps are not accurate. From the comparison of the band structure of $Cs_2AgSbCl_6$ at the PBE and GW levels of theory, it can be estimated that in these compounds, the bandgap is underestimated with PBE by about 0.8 eV.

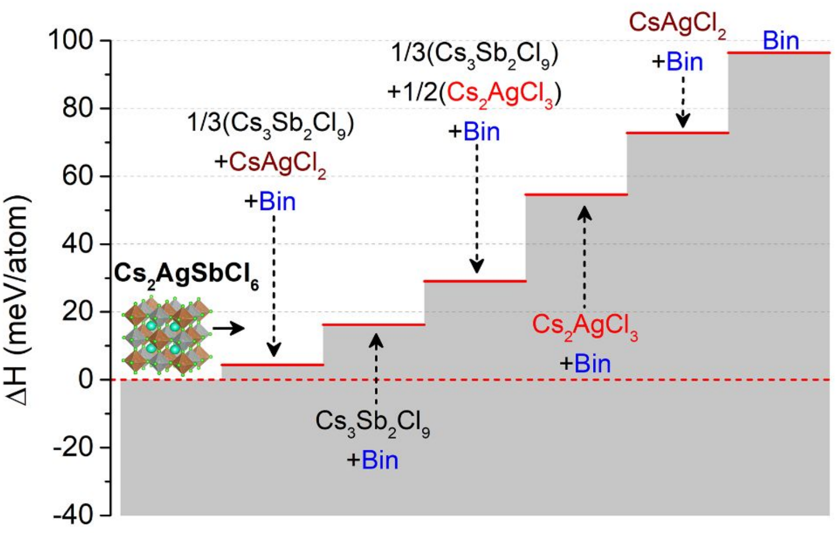

Figure S28. Decomposition enthalpies of $Cs_2AgSbCl_6$ into different ternary and binary chlorides (labeled as Bin).

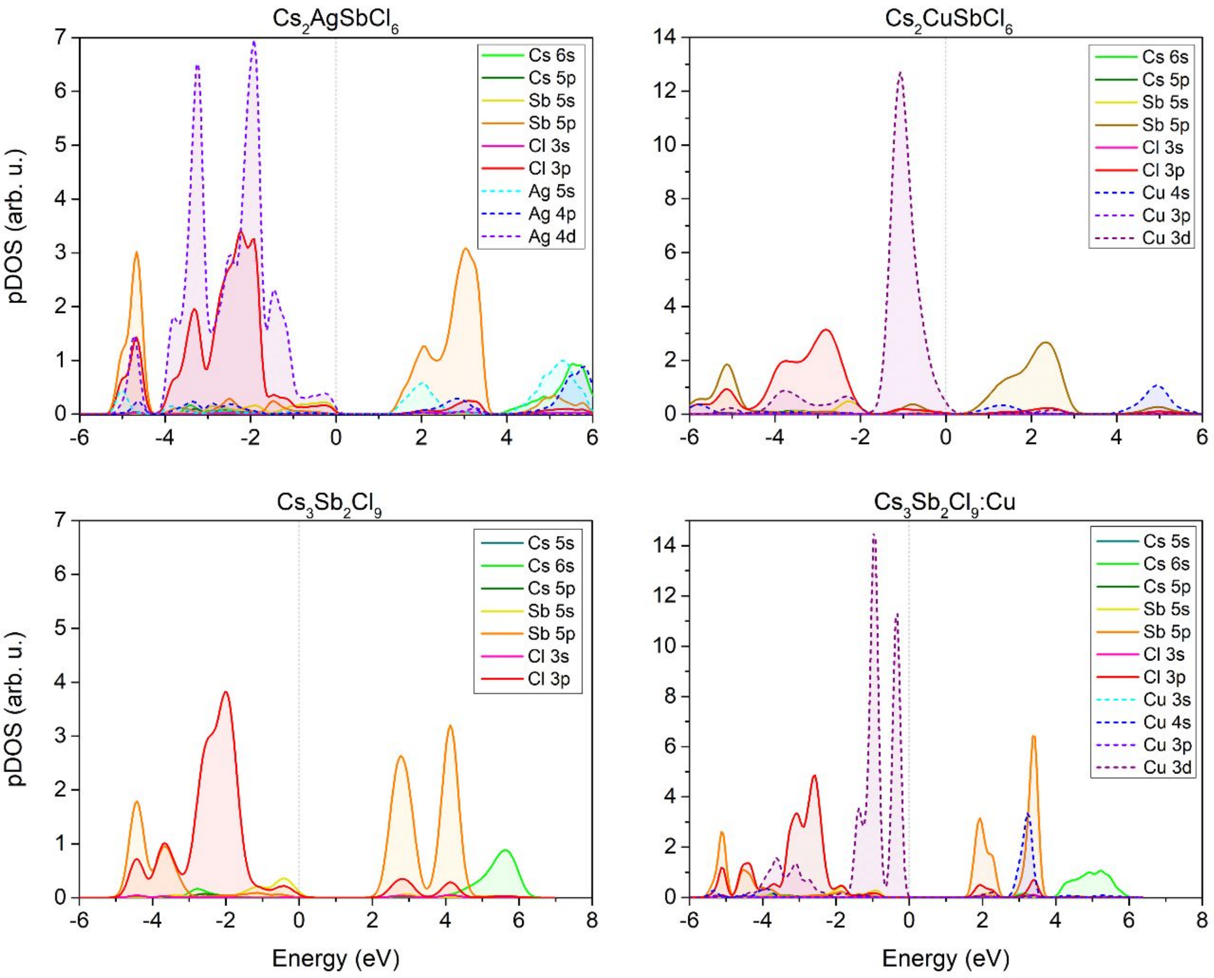

Figure S29. pDOS of $Cs_2CuSbCl_6$, $Cs_2AgSbCl_6$, trigonal $Cs_3Sb_2Cl_9$ and theoretical $Cs_3Sb_2Cl_9$:Cu at the PBE level. The valence band maximum is aligned to zero energy (faint gray line) in all plots.

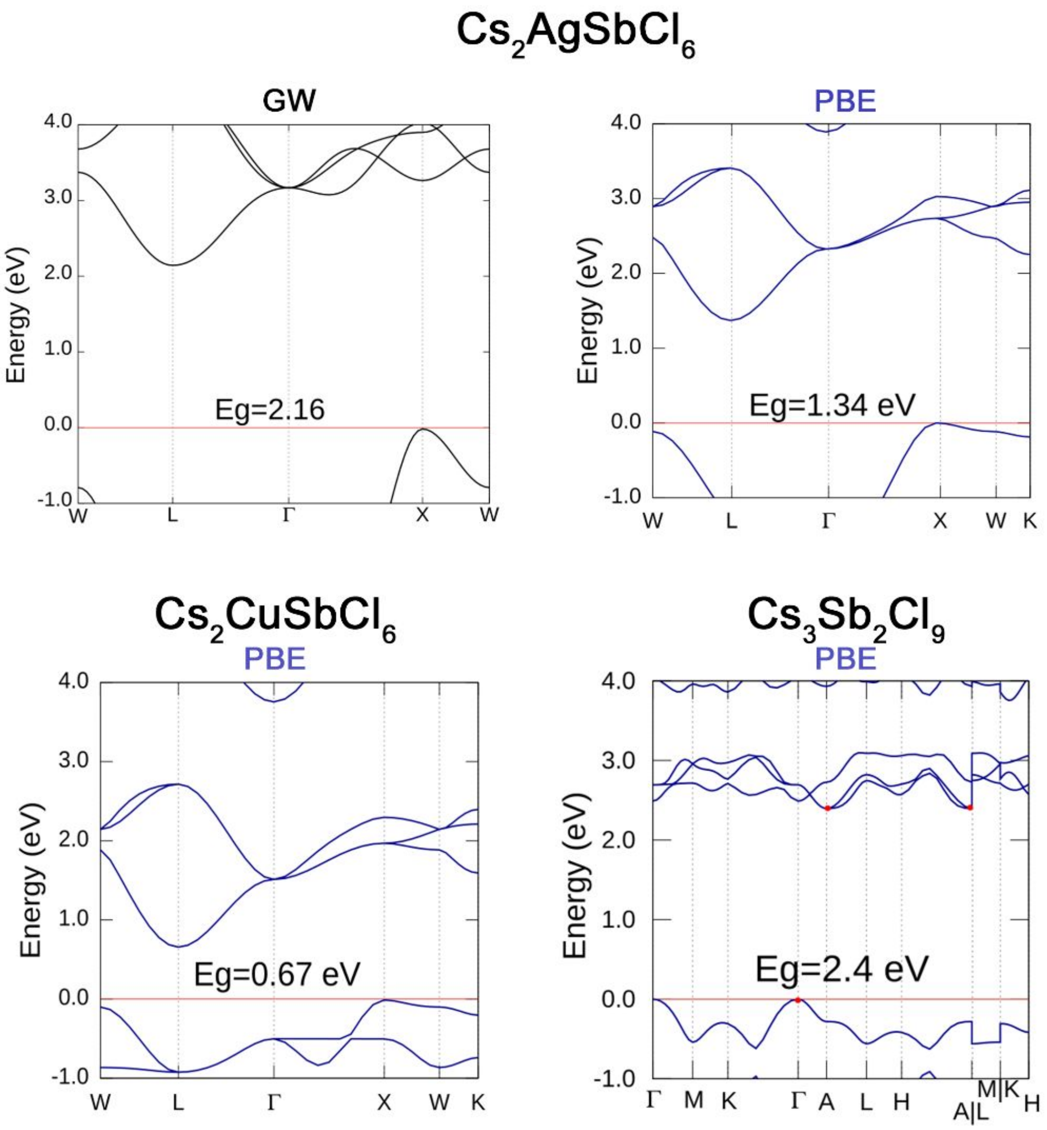

Figure S30. Electronic band structures of $Cs_2AgSbCl_6$ (at the PBE and GW levels of theory, upper panels), and of $Cs_2CuSbCl_6$ and trigonal $Cs_3Sb_2Cl_9$ (at the PBE level, lower panels).

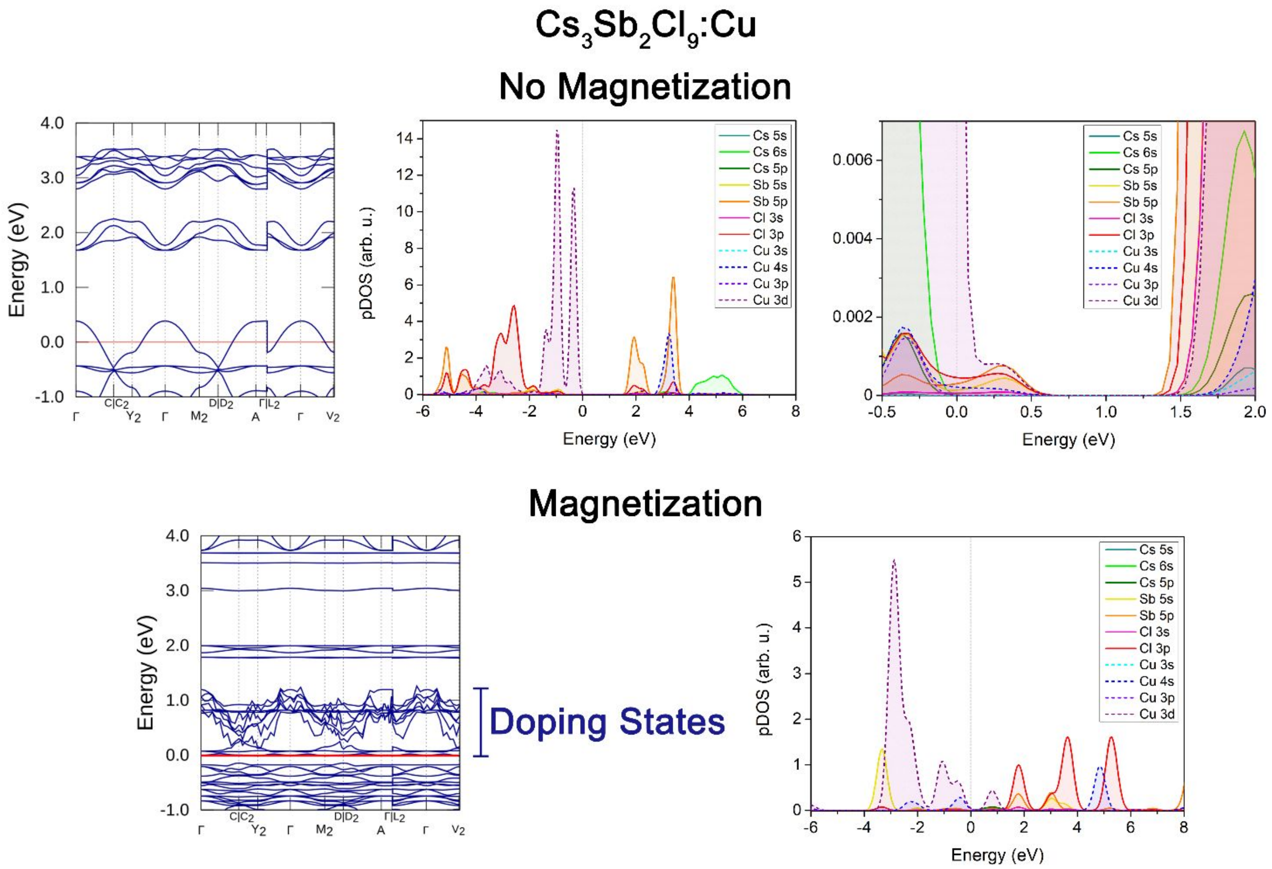

Figure S31. Electronic band structures and pDOS of $Cs_3Sb_2Cl_9$:Cu ($Cs_4CuSb_4Cl_{18}$) at the PBE level without magnetization (above) and with magnetization (below) due to the unpaired electron at the Cu^{2+} site. The red line in the band structure represents the Fermi energy.

Assessment of the beam-induced radiation damage at ID26

For the X-ray absorption measurements carried out at ID26, considering the higher interaction cross-section at relatively low energies and the focused beam, an *ad hoc* procedure was followed to assess radiation damage: each sample was divided into a grid of around 100 spots, and several scans were carried out as needed to increase the statistics. Repeated 10-second spectra were acquired on each spot, averaging separately all the first spectra on each spot ("fresh") and all the subsequent spectra on each spot ("damaged"). It is found that the variation between fresh and damaged spectra is negligible compared to the variation between different compounds.

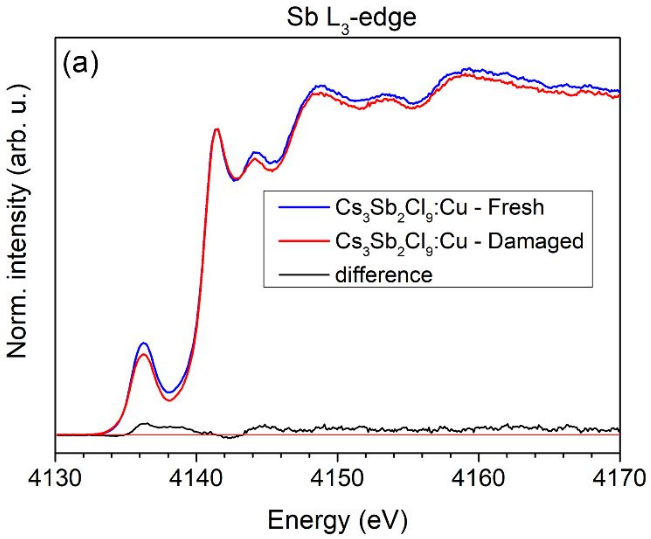

Figure S32. XANES spectra of $Cs_3Sb_2Cl_9$:Cu: fresh and irradiated sample (blue and red, respectively) and their difference (black), for a) Sb L_3-edge and b) Cl K-edge.

References

(1) Zhou, W.; Han, P.; Zhang, X.; Zheng, D.; Yang, S.; Yang, Y.; Luo, C.; Yang, B.; Hong, F.; Wei, D.; Lu, R.; Han, K. Lead-Free Small-Bandgap $Cs_2CuSbCl_6$ Double Perovskite Nanocrystals. *J. Phys. Chem. Lett.* **2020**, 11, 6463–6467, doi:10.1021/acs.jpclett.0c01968.

(2) Yildirim, B.; Cole, J. M. Bayesian Particle Instance Segmentation for Electron Microscopy Image Quantification. *J. Chem. Inf. Model.* **2021**, 61, 1136–1149, doi:10.1021/acs.jcim.0c01455.

(3) Momma, K.; Izumi, F. VESTA: A Three-Dimensional Visualization System for Electronic and Structural Analysis. *J. Appl. Crystallogr. - J APPL CRYST* **2008**, 41, 653–658, doi:10.1107/S0021889808012016.

(4) Yamatera, H.; Nakatsu, K. The Crystal Structure of $Cs_3Fe_2Cl_9$ and of $Cs_3Sb_2Cl_9$. *Bull. Chem. Soc. Jpn.* **1954**, 27, 244, doi:10.1246/bcsj.27.244.

(5) Kihara, K.; Sudo, T. The Crystal Structures of β-$Cs_3Sb_2Cl_9$ and of $Cs_3Bi_2Cl_9$. *Acta Crystallogr. Sect. B* **1974**, 30, 1088–1093, doi:10.1107/S0567740874004316.

(6) Morosin, B.; Lingafelter, E. C. THE CONFIGURATION OF THE TETRACHLOROCUPRATE(II) ION. *J. Phys. Chem.* **1961**, 65, 50–51, doi:10.1021/j100819a015.

(7) Topas-R, V3.0, **2005**.

(8) Klementev, K. V. Extraction of the Fine Structure from X-Ray Absorption Spectra. *J. Phys. D. Appl. Phys.* **2001**, 34, 209, doi:10.1088/0022-3727/34/2/309.

(9) Rehr, J. J.; Kas, J. J.; Vila, F. D.; Prange, M. P.; Jorissen, K. Parameter-Free Calculations of X-Ray Spectra with FEFF9. *Phys. Chem. Chem. Phys.* **2010**, 12, 5503–5513, doi:10.1039/B926434E.

(10) Bunău, O.; Joly, Y. Self-Consistent Aspects of x-Ray Absorption Calculations. *J. Phys. Condens. Matter* **2009**, 21, 345501, doi:10.1088/0953-8984/21/34/345501.

(11) Giannozzi, P.; Baseggio, O.; Bonfà, P.; Brunato, D.; Car, R.; Carnimeo, I.; Cavazzoni, C.; de Gironcoli, S.; Delugas, P.; Ferrari Ruffino, F.; Ferretti, A.; Marzari, N.; Timrov, I.; Urru, A.; Baroni, S. Quantum ESPRESSO toward the Exascale. *J. Chem. Phys.* **2020**, 152, 154105, doi:10.1063/5.0005082.

(12) Giannozzi, P.; Andreussi, O.; Brumme, T.; Bunau, O.; Buongiorno Nardelli, M.; Calandra, M.; Car, R.; Cavazzoni, C.; Ceresoli, D.; Cococcioni, M.; Colonna, N.; Carnimeo, I.; Dal Corso, A.; de Gironcoli, S.; Delugas, P.; DiStasio, R. A. J.; Ferretti, A.; Floris, A.; Fratesi, G.; Fugallo, G.; Gebauer, R.; Gerstmann, U.; Giustino, F.; Gorni, T.; Jia, J.; Kawamura, M.; Ko, H.-Y.; Kokalj, A.; Küçükbenli, E.; Lazzeri, M.; Marsili, M.; Marzari, N.; Mauri, F.; Nguyen, N. L.; Nguyen, H.-V.; Otero-de-la-Roza, A.; Paulatto, L.; Poncé, S.; Rocca, D.; Sabatini, R.; Santra, B.; Schlipf, M.; Seitsonen, A. P.; Smogunov, A.; Timrov, I.; Thonhauser, T.; Umari, P.; Vast, N.; Wu, X.; Baroni, S. Advanced Capabilities for Materials Modelling with Quantum ESPRESSO. *J. Phys. Condens. Matter* **2017**, 29, 465901, doi:10.1088/1361-648X/aa8f79.

(13) Giannozzi, P.; Baroni, S.; Bonini, N.; Calandra, M.; Car, R.; Cavazzoni, C.; Ceresoli, D.; Chiarotti, G. L.; Cococcioni, M.; Dabo, I.; Dal Corso, A.; de Gironcoli, S.; Fabris, S.; Fratesi, G.; Gebauer, R.; Gerstmann, U.; Gougoussis, C.; Kokalj, A.; Lazzeri, M.; Martin-Samos, L.; Marzari, N.; Mauri, F.; Mazzarello, R.; Paolini, S.; Pasquarello, A.; Paulatto, L.; Sbraccia, C.; Scandolo, S.; Sclauzero, G.; Seitsonen, A. P.; Smogunov, A.; Umari, P.; Wentzcovitch, R. M. QUANTUM ESPRESSO: A Modular and Open-Source Software Project for Quantum Simulations of Materials. *J. Phys. Condens. Matter* **2009**, 21, 395502, doi:10.1088/0953-8984/21/39/395502.

(14) Kresse, G.; Joubert, D. From Ultrasoft Pseudopotentials to the Projector Augmented-Wave Method. *Phys. Rev. B* **1999**, 59, 1758–1775, doi:10.1103/PhysRevB.59.1758.

(15) Perdew, J. P.; Burke, K.; Ernzerhof, M. Generalized Gradient Approximation Made Simple. *Phys. Rev. Lett.* **1996**, 77, 3865–3868, doi:10.1103/PhysRevLett.77.3865.

(16) Marini, A.; Hogan, C.; Grüning, M.; Varsano, D. Yambo: An Ab Initio Tool for Excited State Calculations. *Comput. Phys. Commun.* **2009**, 180, 1392–1403, doi:10.1016/j.cpc.2009.02.003.

(17) Sangalli, D.; Ferretti, A.; Miranda, H.; Attaccalite, C.; Marri, I.; Cannuccia, E.; Melo, P.; Marsili, M.; Paleari, F.; Marrazzo, A.; Prandini, G.; Bonfà, P.; Atambo, M. O.; Affinito, F.; Palummo, M.; Molina-Sánchez, A.; Hogan, C.; Grüning, M.; Varsano, D.; Marini, A. Many-Body Perturbation Theory Calculations Using the Yambo Code. *J. Phys. Condens. Matter* **2019**, 31, 325902, doi:10.1088/1361-648X/ab15d0.

(18) Dal Corso, A. Pseudopotentials Periodic Table: From H to Pu. *Comput. Mater. Sci.* **2014**, 95, 337–350, doi:10.1016/j.commatsci.2014.07.043.

(19) Perdew, J. P.; Ruzsinszky, A.; Csonka, G. I.; Vydrov, O. A.; Scuseria, G. E.; Constantin, L. A.; Zhou, X.; Burke, K. Restoring the Density-Gradient Expansion for

Exchange in Solids and Surfaces. *Phys. Rev. Lett.* **2008**, 100, 136406, doi:10.1103/PhysRevLett.100.136406.

(20) Macias-Pinilla, D. F.; Giannici, F. Computational Insights into the Structural and Optical Properties of Ag-Based Halide Double Perovskites. *ACS Appl. Mater. Interfaces* **2025**, 17, 20501–20518, doi:10.1021/acsami.4c22290.

(21) Xiao, Z.; Du, K.-Z.; Meng, W.; Mitzi, D. B.; Yan, Y. Chemical Origin of the Stability Difference between Copper(I)- and Silver(I)-Based Halide Double Perovskites. *Angew. Chemie Int. Ed.* **2017**, 56, 12107–12111, doi:10.1002/anie.201705113.

(22) Gao, W.; Xia, W.; Wu, Y.; Ren, W.; Gao, X.; Zhang, P. Quasiparticle Band Structures of CuCl, CuBr, AgCl, and AgBr: The Extreme Case. *Phys. Rev. B* **2018**, 98, 45108, doi:10.1103/PhysRevB.98.045108.

(23) Leon, D. A.; Ferretti, A.; Varsano, D.; Molinari, E.; Cardoso, C. Efficient Full Frequency GW for Metals Using a Multipole Approach for the Dielectric Screening. *Phys. Rev. B* **2023**, 107, 155130, doi:10.1103/PhysRevB.107.155130.

(24) Jiang, H.; Gomez-Abal, R. I.; Rinke, P.; Scheffler, M. First-Principles Modeling of Localized d States with the GW@LDA + U Approach. *Phys. Rev. B* **2010**, 82, 45108, doi:10.1103/PhysRevB.82.045108.
